# Integrated analysis of single-cell and bulk RNA-sequencing data reveals the prognostic value and molecular function of THSD7A in gastric cancer

**DOI:** 10.18632/aging.205158

**Published:** 2023-10-30

**Authors:** Kaiyu Shen, Binyu Chen, Liu Yang, Wencang Gao

**Affiliations:** 1 The Second Clinical Medical College of Zhejiang Chinese Medical University, Hangzhou 310053, China; 2 Department of Oncology, The Second Affiliated Hospital, Zhejiang Chinese Medical University, Hangzhou 310005, China

**Keywords:** THSD7A, gastric cancer, single-cell RNA sequencing, diagnosis, prognosis

## Abstract

The biological role and prognostic value of thrombospondin domain-containing 7A (THSD7A) in gastric cancer remain unclear. Our purpose was to determine the molecular mechanisms underlying the functioning of THSD7A and its prognostic value in gastric cancer. Gastric cancer-associated single cell and bulk RNA sequencing data obtained from two databases, were analyzed. We used bulk RNA sequencing to examine the differential expression of THSD7A in gastric cancer and normal gastric tissues and explored the relationship between THSD7A expression and clinicopathological characteristics. Kaplan–Meier survival and Cox analyses revealed the prognostic value of THSD7A. Gene set enrichment and immune infiltration analyses were used to determine the cancer-promoting mechanisms of THSD7A and its effect on the immune microenvironment. We explored the relationship between THSD7A expression and sensitivity of anti-tumor drugs and immune checkpoint levels. Biological functions of THSD7A were validated at single-cell and *in vitro* levels. THSD7A expression was significantly increased in gastric cancer samples. High THSD7A expression was associated with poor clinical phenotypes and prognoses. Cox analysis showed that THSD7A was an independent risk factor for patients with gastric cancer. Enrichment analysis suggested that epithelial-mesenchymal transition and inflammatory responses may be potential pro-cancer mechanisms of THSD7A. Upregulation of THSD7A promoted infiltration by M2 macrophages and regulatory T cells. High THSD7A expression suppressed the sensitivity of patients with gastric cancer to drugs, such as 5-fluorouracil, bleomycin, and cisplatin, and upregulated immune checkpoints, such as HAVCR2, PDCD1LG2, TIGIT, and CTLA4. At the single cell level, *THSD7A* was an endothelial cell-associated gene and endothelial cells overexpressing THSD7A showed unique pro-oncogenic effects. *In vitro* experiments confirmed that THSD7A was overexpressed in gastric cancer samples and cells, and that knocking out THSD7A significantly inhibited gastric cancer cell proliferation and invasion. THSD7A overexpression may be a unique prognostic marker and therapeutic target in gastric cancer. Therefore, our study provides a new perspective on the precise treatment of gastric cancer.

## INTRODUCTION

Gastric cancer is a gastrointestinal disease with high molecular and phenotypical heterogeneity. Gastric cancer involves multiple genes, factors, and stages; however, its specific molecular mechanisms remain unclear [1]. Due to yet-unknown causes and limited preventive measures, the five-year survival rate of gastric cancer remains less than 30% [2], reducing the quality of life of patients and increasing the economic burden. The continuing increase in new cases makes gastric cancer a serious public health issue worldwide. At present, early detection of gastric cancer is mainly based on gastroscopy, an unpopular and invasive test. The value of traditional biomarkers, such as carcinoembryonic antigen and carbohydrate antigen 19-9, in the prognosis of patients with gastric cancer as well as in predicting the efficacy of neoadjuvant therapy remains controversial [3]. Currently, a large volume of data pertaining to potential biomarkers of gastric cancer, such as anti-*Helicobacter pylori* antibodies, pepsinogen, and gastrin 17, are available [4, 5]. Biomarkers have also been utilized to treat gastric cancer. However, inconsistencies in examination methods, standards, and quality control techniques have led to the realization that these markers need to be further validated via large-scale clinical studies. This situation is further exacerbated by the decreased efficacy of traditional first-line therapies, increased drug resistance to drugs aimed at gastric cancer, and the complexities associated with signaling pathways within the tumor microenvironment (TME), all of which indicate the necessity for developing effective therapeutic targets for gastric cancer.

Thrombospondin type 1 domain containing 7A (THSD7A), a transmembrane protein with a relative molecular mass of approximately 250,000 Da, is commonly expressed on the surface of glomerular podocytes in humans and rodents [6]. The extracellular region of THSD7A consisting of the thrombospondin type 1 domain, which is tagged by the histamine of thrombospondin 1 and complement component 6, interacts with proteases and the extracellular matrix (ECM) [7–9]. Studies indicate that the main anti-THSD7A antibody in patients with idiopathic membranous nephropathy is IgG4, which targets the N-termini of autoantigens, and that the expression of IgG4 is positively correlated with the severity of proteinuria [10]. THSD7A reportedly plays an important role in diseases such as osteoporosis and tumorigenesis [11, 12]. Hoxha et al. found that THSD7A-positive patients with membranous nephropathy, whose tumor foci exhibited significantly elevated expression levels of THSD7A mRNA, showed an increased risk for cancer development [13]. In addition, Stahl et al. reported that THSD7A-based staining was enhanced in a variety of solid tumors, such as colorectal, prostate, and renal cancers, and that immunohistochemical analysis of tissue microarrays indicated the prognostic potential of THSD7A [12].

However, reports on the correlation between THSD7A expression and gastric cancer remain scant, indicating the need for further exploration. In this study, we investigated whether THSD7A expression shows prognostic value in gastric cancer and examined the possible molecular mechanisms underlying the role of THSD7A in gastric cancer via bioinformatics and *in vitro* experiments, with the expectation that our findings would provide new strategies for exploring targeted therapy for gastric cancer.

## MATERIALS AND METHODS

### Data acquisition

Transcriptomic data and paired clinical data containing 32 normal samples and 375 gastric cancer samples were obtained from The Cancer Genome Atlas (TCGA) database (https://cancergenome.nih.gov/), in which gastric cancer samples with incomplete clinical information were excluded. The GSE27342 (normal=80, tumor=80), GSE29272 (normal=134, tumor=134), GSE54129 (normal=21, tumor=111), and GSE65801 (normal=32, tumor=32) from the Gene Expression Omnibus (GEO) (www.ncbi.nlm.nih.gov/geo) database provided the standardized transcriptomic data of normal gastric tissues and gastric cancer tissues. In addition, we obtained 10 gastric cancer samples from GSE167297, including the scRNA-seq data from five superficial gastric cancer, five deep gastric cancer, and four para-cancer samples [14].

### Expression of THSD7A in gastric cancer

The differential expression of THSD7A in gastric cancer and normal gastric tissues was analyzed based on the UALCAN database (https://ualcan.path.uab.edu/) [15] and validated in the external data of GEO. Meanwhile, we also explored the expression level of THSD7A associated with different clinicopathological characteristics.

### Prognostic significance of THSD7A

To identify the prognostic value of THSD7A in gastric cancer, we analyzed the correlation between THSD7A expression and OS and DFS using The Gene Expression Profiling Interactive Analysis (GEPIA) database (http://gepia.cancer-pku.cn/index.html). Subsequently, the Kaplan–Meier Plotter tool (https://kmplot.com/analysis/) was used to validate the association between THSD7A (check ID: 213894_at) and the OS, FPS, and PPS of patients with gastric cancer. To test whether THSD7A was an independent prognostic factor, we integrated the clinical information of the TCGA-STAD cohort with the transcriptomic data of THSD7A for univariate and multivariate Cox analyses. We also constructed a THSD7A-nomogram for predicting the survival probability of patients with gastric cancer at 1, 3, and 5 years.

### Functional enrichment analysis

In the gene set enrichment analysis (GSEA), we can obtain the gene expression profile data by setting specific functional gene sets [16]. The GSEA software (version 4.1.0) was used to analyze the functional signaling pathway of high- and low-THSD7A expression groups in the TCGA-STAD dataset, with a permutation test parameter of 1000 and the gene set parameters being “h.all.v2022.1.Hs.symbols.gmt” and “c2.cp.kegg.v2022.1.Hs.symbols.gmt”. The statistical significance threshold was set at NOM P-value <0.05.

### Analysis of THSD7A and tumor immune cells

As previously described [17], we used the CIBERSORT algorithm to evaluate the infiltration abundance of 22 types of leukocytes in each TCGA-STAD sample. The Spearman correlation test confirmed the correlation between THSD7A levels and infiltration of tumor immune cells (TICs).

### Drug sensitivity, immune checkpoints, effectiveness of immunotherapy, and tumor mutation burden

To fully understand the predictive role of THSD7A in anti-tumor treatment of gastric cancer, we first divided the TCGA-STAD cohort into high- and low-THSD7A expression groups according to the median THSD7A expression value. Using the Genomics of Drug Sensitivity in Cancer database (https://www.cancerrxgene.org/) [18], with the help of the “pRRophetic” and “limma” packages, we analyzed the differences in common chemotherapeutic drugs and immune checkpoints between the high- and low-THSD7A expression groups. Based on the Tumor Immune Dysfunction and Exclusion (TIDE) (http://tide.dfci.harvard.edu/login/) to compare the risk of the efficacy of immunotherapy between gastric cancer patients with high and low THSD7A expression. Additionally, we examined the correlations between THSD7A expression and Tumor Mutation Burden (TMB) score. We also evaluated the differences in survival among individuals with gastric cancer who had various THSD7A expression and TMB scores.

### Single-cell RNA sequence analysis

The single-cell RNA sequence (scRNA-seq) analysis provides further insight into the role of THSD7A in subsets of gastric cancer cells. The “Seurat” package was used to process the scRNA-seq data of the gastric cancer and para-cancer cells in this study. The imported single-cell gene expression profiles were converted into analysis objects using the “CreateSeuratObject” function, and low-quality cells were discarded based on a high percentage of mitochondrial genes (>20%) and the number of expressed genes in the cells being <500. After normalizing the data using the “NormaliseData” function, we selected 2000 highly variable genes for principal component analysis and clustered the cell subgroups at a resolution of 0.5 using the *t*-SNE algorithm. The “FindAllMarkers” function was used to calculate the marker genes in each cell cluster. We annotated the cell types with the help of the CellMarker database (http://bio-bigdata.hrbmu.edu.cn/CellMarker/) [19]. The “PercentageFeatureSet” function was used to analyze THSD7A expression in THSD7A-related cells and to define THSD7A-related cells as THSD7A (+)-related cells and THSD7A (-)-related cells using the percentage of THSD7A expression of >0 and =0, respectively. Subsequently, the “monocle2” package was used to analyze the developmental trajectory of THSD7A-related cells during gastric cancer progression. The function “differentialGeneTest” was employed to ascertain the differential genes present in THSD7A (+) endothelial cells and THSD7A (-) endothelial cells. Subsequently, the genes that exhibited differential expression with a significant q-value < 0.01 were utilized as input for the “reduceDimension” function. The function was configured with the following parameters: max_components = 3, num_dim = 4, and reduction_method = “DDRTree”. We employed dimensionality reduction clustering and subsequently utilized the “orderCells” function to estimate cell trajectories. To further explore the biological mechanisms involving THSD7A at the single-cell level in gastric cancer, we calculated the gene clusters along the cell developmental trajectory and performed enrichment analysis for the gene clusters with consistent THSD7A expression patterns. The “CellChat” package [20] was used to identify the interactions between THSD7A-related cells and other cells in the TME.

### Immunohistochemistry procedure

Tissue microarrays (array no. ZL-StmA961) containing 48 gastric cancer samples and paired para-cancer samples were purchased from Shanghai Weiao Biotech Co., Ltd. (Shanghai, China). The primary antibody for THSD7A was a rabbit polyclonal primary antibody (HPA000923; Sigma Aldrich, St. Louis, MO, USA), diluted at a ratio of 1:100. After dewaxing and hydration, the tissue microarrays were incubated in 3% hydrogen peroxide for 10 min, followed by antigen repair with sodium citrate solution for 10 min, blocking at room temperature for 120 min, and incubation with anti-THSD7A antibody overnight at 4° C. Finally, 3,3′-diaminobenzidine staining was used to examine THSD7A expression, and re-staining was performed with hematoxylin for 5 min. The expression of THSD7A protein was quantified using the Image Pro Plus (version 6.0; Media Cybernetics, Rockville, MD, USA) software, and the quantification criterion was density mean = density sum/area sum, with larger values indicating stronger staining [21].

### Cell culture

One normal gastric cell line (GES-1) and three gastric cancer cell lines (MKN-45, HGC-27, and BGC-823) were purchased from Hangzhou Frieden Biotechnology Co., Ltd. (Hangzhou, China). The MKN-45 and BGC-823 cells were cultured in RPMI-1640 medium, whereas the GES-1 and HGC-27 cells were cultured in the Dulbecco’s modified Eagle’s medium containing 10% fetal bovine serum. All cells were cultured at 37° C under a 5% CO_2_ environment.

### Quantitative reverse transcription PCR

To quantify THSD7A expression in the normal gastric cell line and gastric cancer cell lines, total RNA was extracted from GES-1 and the remaining three gastric cancer cell lines using the TRIzol Reagent. Thereafter, Quantitative Reverse Transcription PCR (qRT-PCR) was performed using SYBR Premix Ex Taq (Code: DRR041A; Takara; Tokyo, Japan) according to the manufacturer’s protocol; β-actin was used as an internal control. The expression level of THSD7A was quantified using the 2^-ΔΔCT^ method. The primers for β-actin were F: 5’-TGGCACCCAGCACAATGAA-3’ and R: 5’-CTAAGTCATAGTCCGCCTAGAAGCA-3’, and those for THSD7A were F: 5’-GTGGAGGGATGGACTACACTG-3’ and R: 5’-TGCCAATCGCAAACTTTGAAAC-3’.

### Cell transfection

Silencing of THSD7A was achieved through the human target gene THSD7A shRNA (purchased from GenePharma Co., Ltd., Suzhou, China) with the following sequences: THSD7A-shRNA1: 5’-AGGAGACGCGGGAAGAATAAA-3’, THSD7A-shRNA2: 5’-ATGCCGACATGTAACATATAA-3’ and THSD7A-shRNA3: 5’-TGTCCTTGTGACAAATATAAT-3’. Lipofectamine 3000 (Invitrogen, Waltham, MA, USA) was used to transfect HGC-27 cells, and those infected for 72 h were used for subsequent analysis.

### Cell proliferation assays

To evaluate the proliferative capacity of gastric cancer cells after THSD7A silencing, THSD7A-shRNA- and NC-shRNA-transfected HGC-27 cells were incubated at 37° C and 5% CO_2_. Cell proliferation was evaluated using CCK-8 (Dojindo, Tokyo, Japan), and the absorbance at 450 nm was measured at 0, 24, 48, and 72 h post-transfection.

### Wound healing test

The above HGC-27 cells were inoculated in 6-well plates at a density of 1×10^5^ cells/well. After the cells adhered and reached 80–100% confluence, they were wounded with a 2 μL sterile pipette tip, washed three times with phosphate-buffered saline, and then cultured in a fresh complete medium for 48 h. The cultures were photographed at 0, 24, and 48 h under a microscope at 100× magnification.

### Cell invasion assays

The cell invasion ability of HGC-27 cells was evaluated using a transwell chamber (Corning Inc., Corning, NY, USA). The transwell apparatus consists of two distinct compartments, namely the upper and lower chambers. The top component is incubated at a temperature of 37° C for a duration of 4-5 hours, resulting in the formation of a gel-like matrix. 2 × 1 0^5^ HGC-27 cells transfected with THSD7A-shRNA and NC-shRNA were placed in the upper chamber, whereas complete medium was placed in the lower chamber. After incubation at 37° C with 5% CO_2_ for 48 h, the upper layer of non-invaded cells was removed with cotton swabs. The HGC-27 cells were subjected to fixation using anhydrous methanol for a duration of 15 minutes. The migration of cells from the upper chamber to the lower chamber was facilitated by the permeability of the membrane and the composition of the interstitial media in both chambers. The non-migratory cells presented in the upper chamber were eliminated, resulting in the retention of just the migrated cells in the lower chamber. The wells should be washed using a solution of 1 × phosphate-buffered saline and the HGC-27 cells were fixed and stained with 4% paraformaldehyde and 0.1% crystal violet, respectively. Subsequently, an additional rinse was performed using a solution of 1 x phosphate-buffered saline. Following this, a cotton swab was utilized to remove all cells that have not moved. The cells were observed under a microscope at 400× magnification and analyzed using the ImageJ software (version 1.8.0.112; National Institutes of Health, Bethesda, MD, USA).

### Colony formation assays

HGC-27 cells transfected with NC-shRNA and THSD7A-shRNA were uniformly seeded onto plates and incubated for a duration of 2 weeks at a temperature of 37° C in a controlled incubation environment. The cell density was maintained at 500 cells/well. The formation and observation of colonies were carried out, followed by the termination of the culture and subsequent removal of the supernatant. This was followed by two rinses in phosphate-buffered saline. The cells were fixed with 4% paraformaldehyde and stained with crystal violet. The cells should be washed in the staining solution at a gentle pace, followed by rinsing with running water and subsequent air drying, followed by quantitative analysis using the ImageJ software (version 1.8.0.112; National Institutes of Health).

### Statistical analysis

All statistical analyses were performed using the R software (version 4.1.0). The Wilcoxon test and Student’s t-test were used to compare the differences between two groups. The Kruskal–Wallis test and One-way ANOVA were used to compare the differences between multiple groups, respectively. The Kaplan–Meier method and log-rank test were used to determine the differences in OS between the high- and low-THSD7A expression groups. The univariate and multivariate Cox regression analyses were used to determine the independent prognostic factors. Spearman’s rank correlation was used for correlation analysis. The graphs were plotted using the R (version 4.1.0) and GraphPad Prism (version 7.0; GraphPad, San Diego, CA, USA) software. The statistical analyses of the cellular studies were conducted using GraphPad Prism software. Student’s t-test was employed to compare two groups, while One-way ANOVA was used to compare three or more groups. The resulting results were reported as the mean ± standard deviation. All experiments were repeated three times. Unless stated otherwise, the differences were considered statistically significant at P<0.05.

### Data availability statement

The scRNA-seq and bulk RNA-seq data from this study are available in the GEO (https://www.ncbi.nlm.nih.gov/geo/) and TCGA (https://portal.gdc.cancer.gov/) databases. Other data can be obtained from the corresponding author upon reasonable request.

## RESULTS

### THSD7A expression is upregulated in gastric cancer

**Figure 1:**
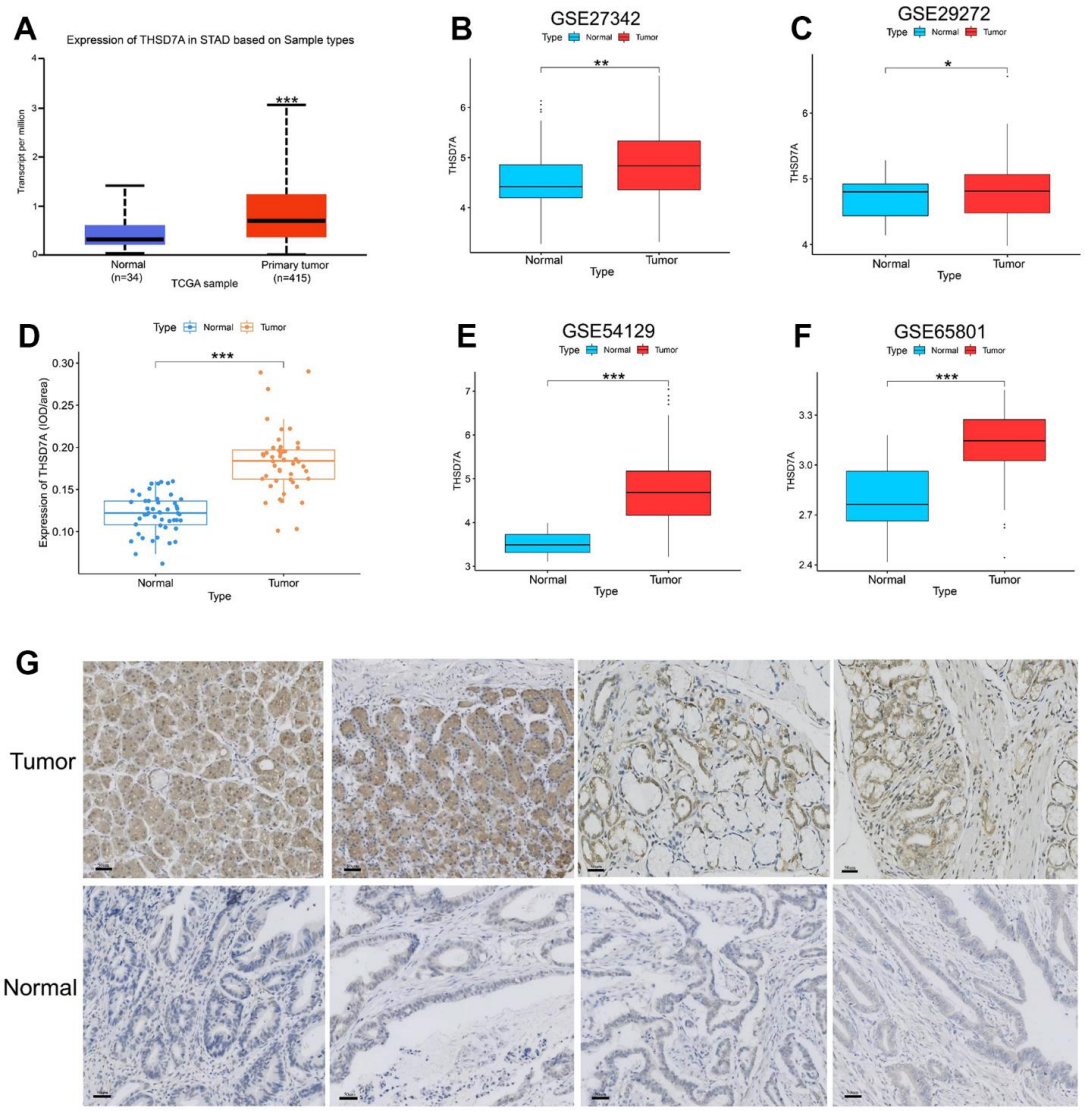
**Analysis of THSD7A expression in the UALCAN database, TCGA database, GEO database, and tissue microarrays.** Differential expression of THSD7A in the UALCAN database (**A**), GSE27342 (**B**), GSE29272 (**C**), GSE54129 (**D**), and GSE65801 (**E**) cohorts. (**F**, **G**) Differential expression of THSD7A in the tissue microarrays of gastric cancer verified via immunohistochemistry (scale bar = 50 μm) (*: P<0.05, **: P<0.01, ***: P<0.001).

**Figure 2:**
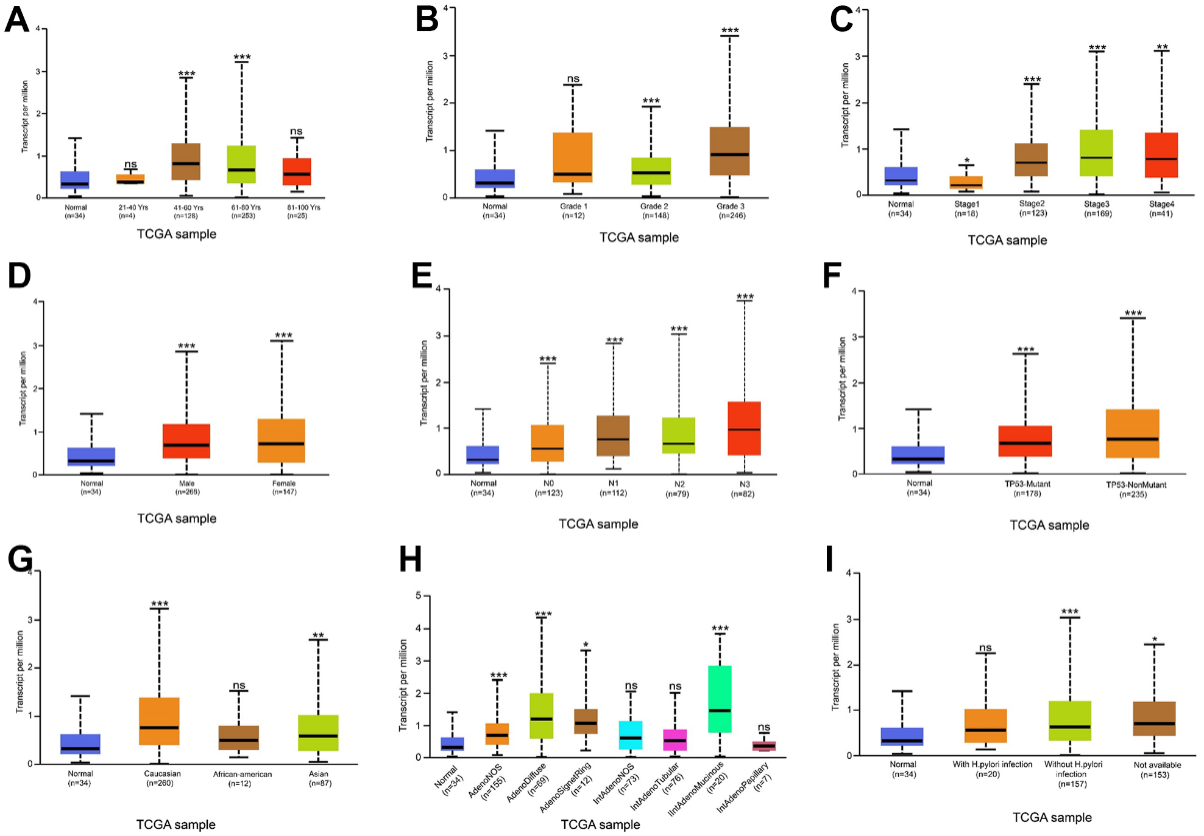
**Relationship between THSD7A expression and clinical characteristics.** Relationship between THSD7A expression and age (**A**), grade (**B**), stage (**C**), sex (**D**), lymph node metastasis stage (**E**), TP53 mutation (**F**), ethnicity (**G**), histological subtypes (**H**) and *Helicobacter pylori* infection (**I**) (*: P<0.05, **: P<0.01, ***: P<0.001, ns: P>0.05). (Normal is the control group.)

Analysis of the data from the UALCAN datasets (cohorts), GSE27342, GSE29272, GSE54129, and GSE65801, revealed that THSD7A is significantly increased in gastric cancer tissues compared with that in normal gastric tissues (P<0.05; Figure 1A–1E). We used immunohistochemistry to analyze THSD7A protein levels in tissue microarrays involving 48 cases of gastric cancer and paired para-cancerous tissues and found that THSD7A protein levels were significantly higher in gastric cancer tissues than in normal gastric tissues (P<0.001; Figure 1F, 1G). THSD7A expression was highest in the 41–60-year age group (P<0.001; Figure 2A). Regarding the grade and TNM stage of gastric cancer, THSD7A was most significantly upregulated in higher grades (grade 3; P<0.001) and higher stages (stage II, III, and IV; P<0.001; Figure 2B, 2C). Compared with that in the normal group, THSD7A expression was higher in patients with gastric cancer of different sexes, those showing positive lymph node metastasis status, and those in the TP53 mutation subgroups (P<0.001; Figure 2D–2F). With respect to race and the characteristics of histological subtypes, the expression of THSD7A was highest in Caucasian patients with gastric cancer (P<0.001) and intestinal adenocarcinoma (mucinous) subtypes (P<0.001; Figure 2G, 2H), respectively. Although the expression of THSD7A in the normal group was lower than that in patients with gastric cancer who were not infected with *Helicobacter pylori* (P<0.001), it was not different from that in *H. pylori*-infected patients with gastric cancer (Figure 2I), which may be attributed to the small sample size.

### Prognostic value of THSD7A and associated clinical factors in gastric cancer

**Figure 3:**
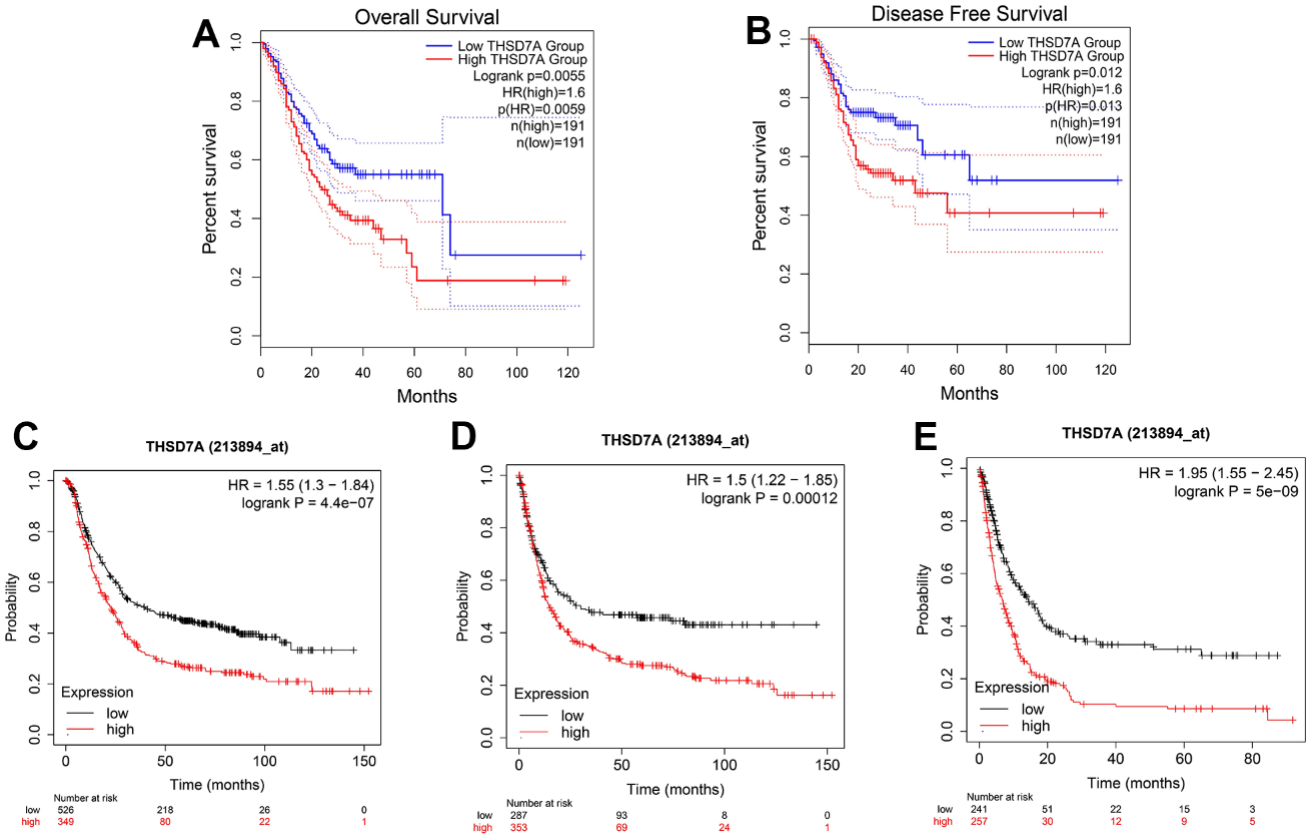
**Survival analysis based on THSD7A expression.** The relationship between high and low THSD7A expression groups in the GEPIA database and OS (**A**) and DFS (**B**). The relationship between the high and low THSD7A expression groups and the OS (**C**), FPS (**D**), and PPS (**E**) using the Kaplan–Meier Plotter tool.

**Figure 4:**
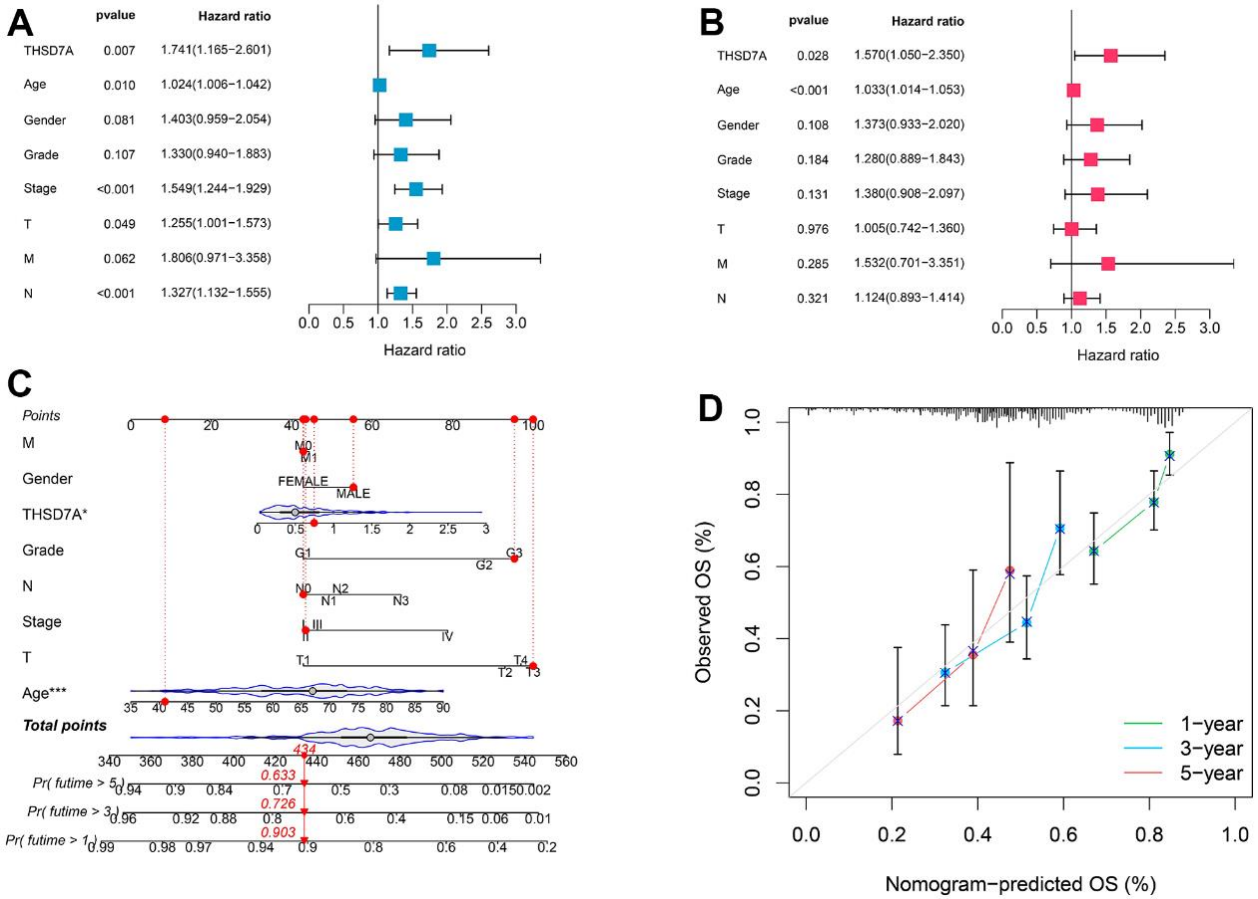
**Prognostic value of THSD7A.** The univariate (**A**) and multivariate (**B**) Cox analyses of the prognostic significance of THSD7A in gastric cancer. (**C**) The THSD7A-nomogram constructed by combining THSD7A expression with clinical characteristics. (**D**) Calibration curve of THSD7A-nomogram.

GEPIA online analysis showed that patients with gastric cancer and high THSD7A expression had shorter overall survival (OS; P<0.05; Figure 3A) and disease-free survival (DFS; P<0.05; Figure 3B), compared with those with low THSD7A expression. The Kaplan–Meier Plotter tool validated the finding that patients with gastric cancer and low THSD7A expression had higher OS (P<0.001), first progression survival (FPS; P<0.001), and post-progression survival (PPS; P<0.001), compared with those with high THSD7A expression (Figure 3C–3E). Combined with the clinical data from TCGA-stomach adenocarcinomas (STAD) cohort, univariate Cox analysis demonstrated that THSD7A expression [hazard ratio (HR) = 1.741, 95% confidence interval (CI): 1.165–2.601, P = 0.007], age (HR = 1.024, CI: 1.006-1.042, P = 0.010), T-stage (HR = 1.225, CI: 1.001-1.573, P = 0.049), and N-stage (HR = 1.327, 95% CI: 1.132-1.555, P < 0.001) were significantly associated with a high-risk ratio (Figure 4A). Multivariate Cox analysis indicated that THSD7A expression (HR = 1.570, 95% CI: 1.050-2.350, P < 0.028) and age (HR = 1.033, 95% CI: 1.014-1.053, P < 0.001) were independent prognostic factors for gastric cancer (Figure 4B). Finally, the nomogram constructed by integrating the THSD7A expression profile and the clinical information of gastric cancer showed that THSD7A expression may play a good predictive role in gastric cancer (Figure 4C), the reliability of which was confirmed by the calibration curve (Figure 4D).

### Functional analysis of THSD7A

**Figure 5:**
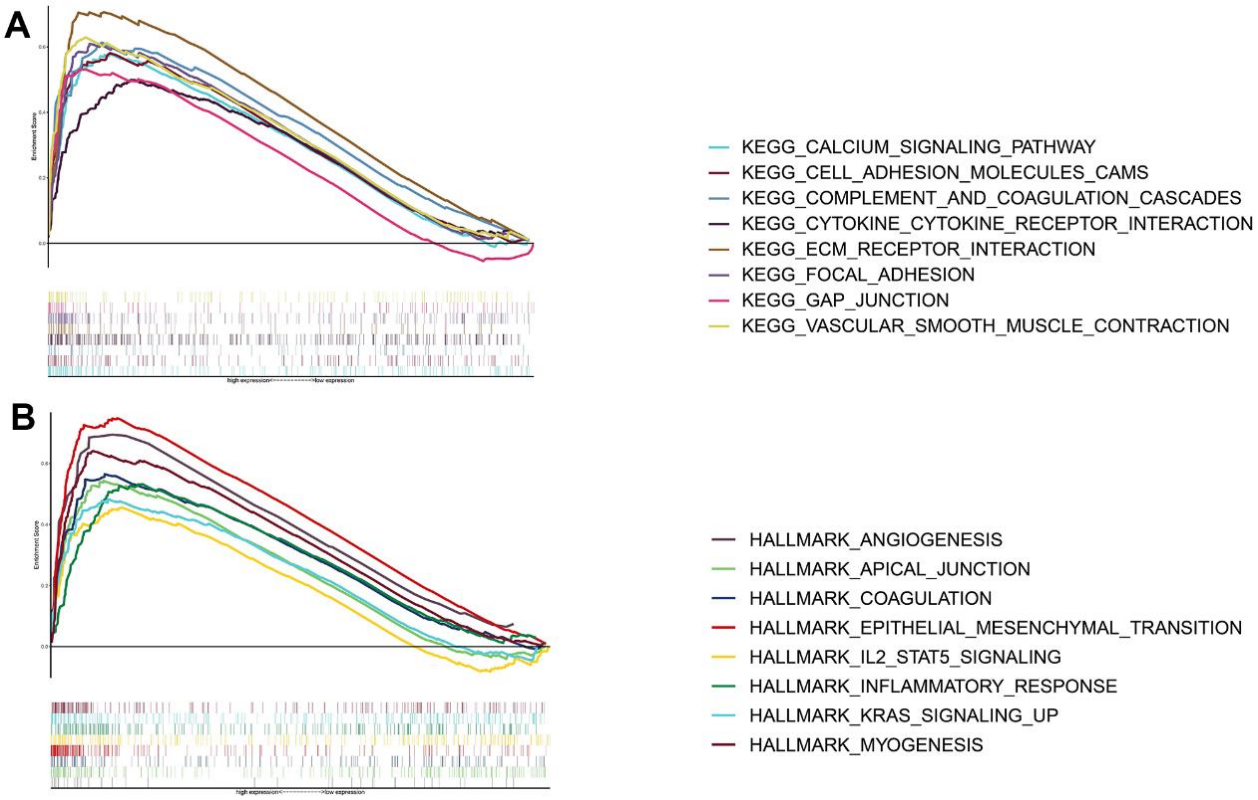
**Functional enrichment analysis of THSD7A.** Functional enrichment analysis based on the KEGG gene set (**A**) and the HALLMARK gene set (**B**).

Based on the GSEA of both gene sets, we explored signaling pathways that THSD7A may possibly be involved in. The results indicated that high THSD7A expression may promote the progression of gastric adenocarcinoma via processes such as epithelial-mesenchymal transition (EMT), the activity of cell adhesion molecules, inflammatory responses, angiogenesis, vascular smooth muscle contraction, the IL2-STAT5 signaling pathway, focal adhesion, and ECM-receptor interaction (Figure 5A, 5B).

### THSD7A expression is correlated with tumor immune cells

**Figure 6:**
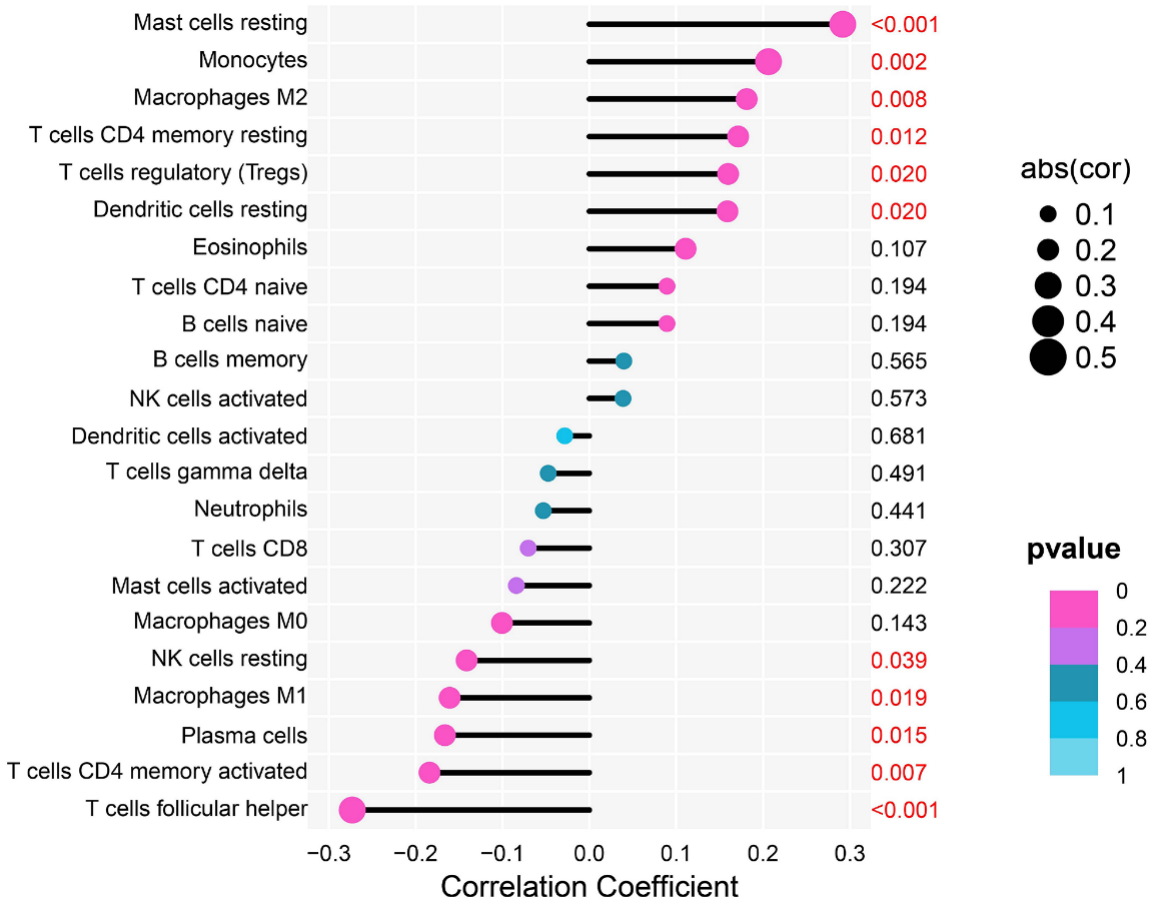
Analysis of the correlation between THSD7A expression and immune cell infiltration.

Oya et al. reported that investigating the gastric cancer-associated TME may aid in identifying new therapeutic targets [22]. Therefore, we performed a correlation analysis between THSD7A and TICs. The results showed that resting mast cells (P<0.001), M2 macrophages (P=0.008), and regulatory T cells (Tregs; P=0.020) were significantly positively correlated with THSD7A expression. In addition, THSD7A expression showed a negative correlation with follicular helper T cells (P<0.001), activated memory CD4^+^ T cells (P=0.007), and M1 macrophages (P=0.019), as shown in Figure 6.

### Half-maximal inhibitory concentration, immune checkpoints, effectiveness of immunotherapy, and tumor mutation burden

**Figure 7:**
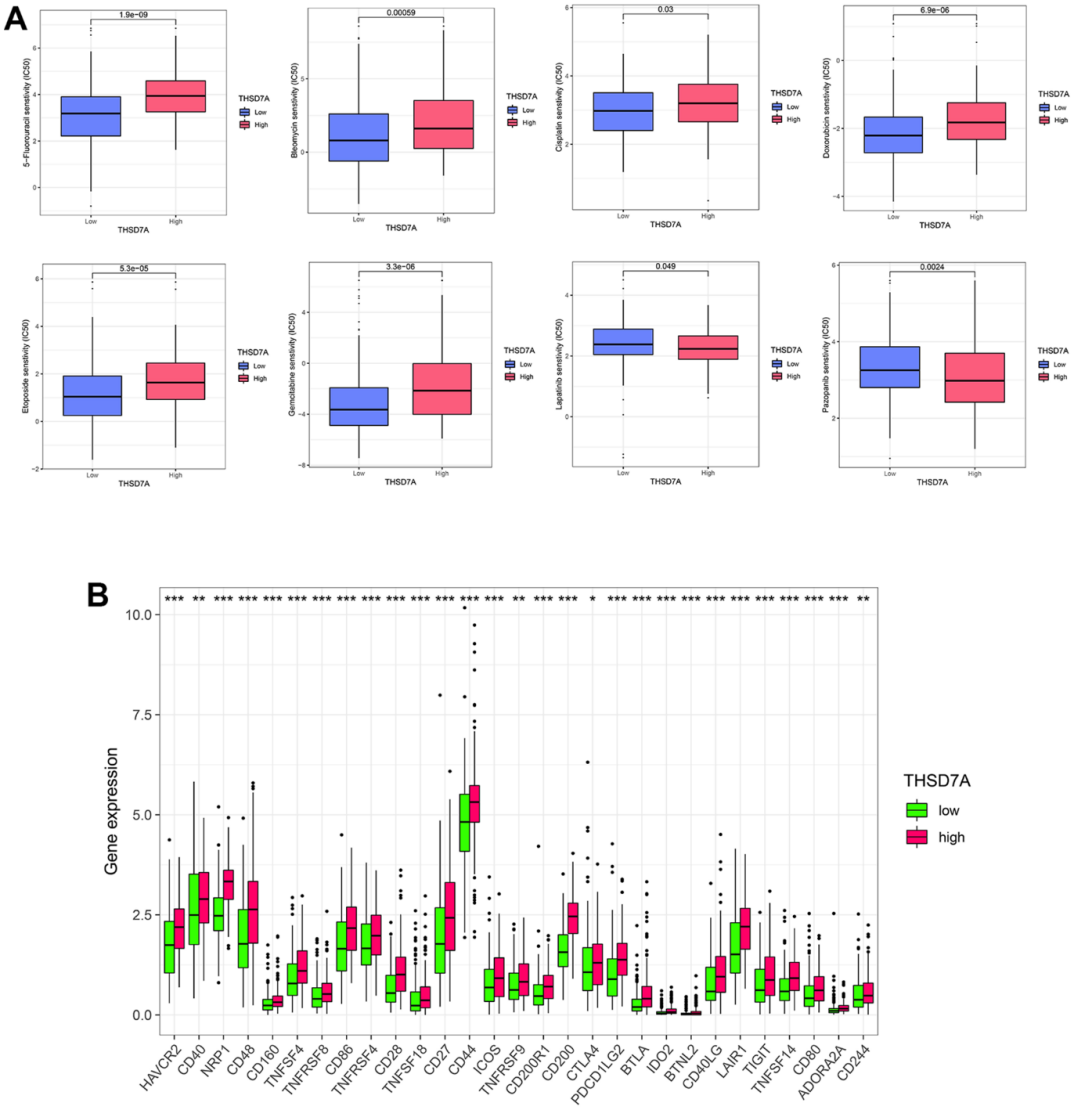
**Analysis of drug sensitivity and immune checkpoints.** (**A**) Correlation between high- and low-THSD7A expression subgroups and the sensitivity to different anti-tumor drugs. (**B**) Expression of immune checkpoints in the high- and low-THSD7A expression groups (*: P<0.05, **: P<0.01, ***: P<0.001).

Next, we evaluated the efficacy of THSD7A expression in predicting the half-maximal inhibitory concentration (IC_50_) values of anti-tumor drugs and checkpoint expression. We found that 5-fluorouracil (P<0.001), bleomycin (P<0.001), cisplatin (P<0.05), doxorubicin (P<0.001), etoposide (P<0.001), and gemcitabine (P<0.001) in the high-THSD7A expression group had higher IC_50_ values than those in the low-THSD7A expression group, whereas lapatinib (P<0.05) and pazopanib (P<0.01) in the high-THSD7A expression group had lower IC_50_ values than those in the low-THSD7A expression group (Figure 7A). In addition, checkpoint molecules such as HAVCR2 (P<0.001), PDCD1LG2 (P<0.001), TIGIT (P<0.001), and CTLA4 (P<0.05) were significantly upregulated in the high-THSD7A expression group (Figure 7B). The TIDE score has been established as a reliable indicator for the prediction of immunotherapy efficacy [23]. The findings of the study indicated that the TIDE score of the group with high expression of THSD7A was notably greater than that of the group with low expression, which indicated that immunotherapy may be less successful in gastric cancer patients with elevated THSD7A expression (Supplementary Figure 1A). Numerous studies have demonstrated that TMB serves as a helpful prognostic indication for tumor immune response.

Patients exhibiting high TMB levels have been observed to get benefits from immunotherapy interventions [24]. The Spearman correlation analysis revealed a negative association between THSD7A and TMB (Supplementary Figure 1B). The findings of our study demonstrated that the TMB in the group with low THSD7A expression was much higher compared to the group with high expression (Supplementary Figure 1C). These results implied that individuals with gastric cancer who exhibited low THSD7A expression might potentially experience greater therapeutic benefits from immunotherapy interventions. Furthermore, survival analysis was performed on various TMB subgroups. The patients who exhibited high TMB demonstrated a more favorable outcome in comparison to those with low TMB (Supplementary Figure 1D). Following this, we conducted a survival study in gastric cancer patients by integrating the expression of TMB and THSD7A. The findings revealed that patients with gastric cancer who belonged to the high TMB group and the low expression THSD7A group had the most favorable prognosis in terms of survival (Supplementary Figure 1E). This finding provided more evidence supporting the notion that individuals with gastric cancer who exhibit reduced THSD7A expression are more susceptible to the effects of immunotherapy.

### Gastric cancer single cell RNA sequencing data analysis

**Figure 8:**
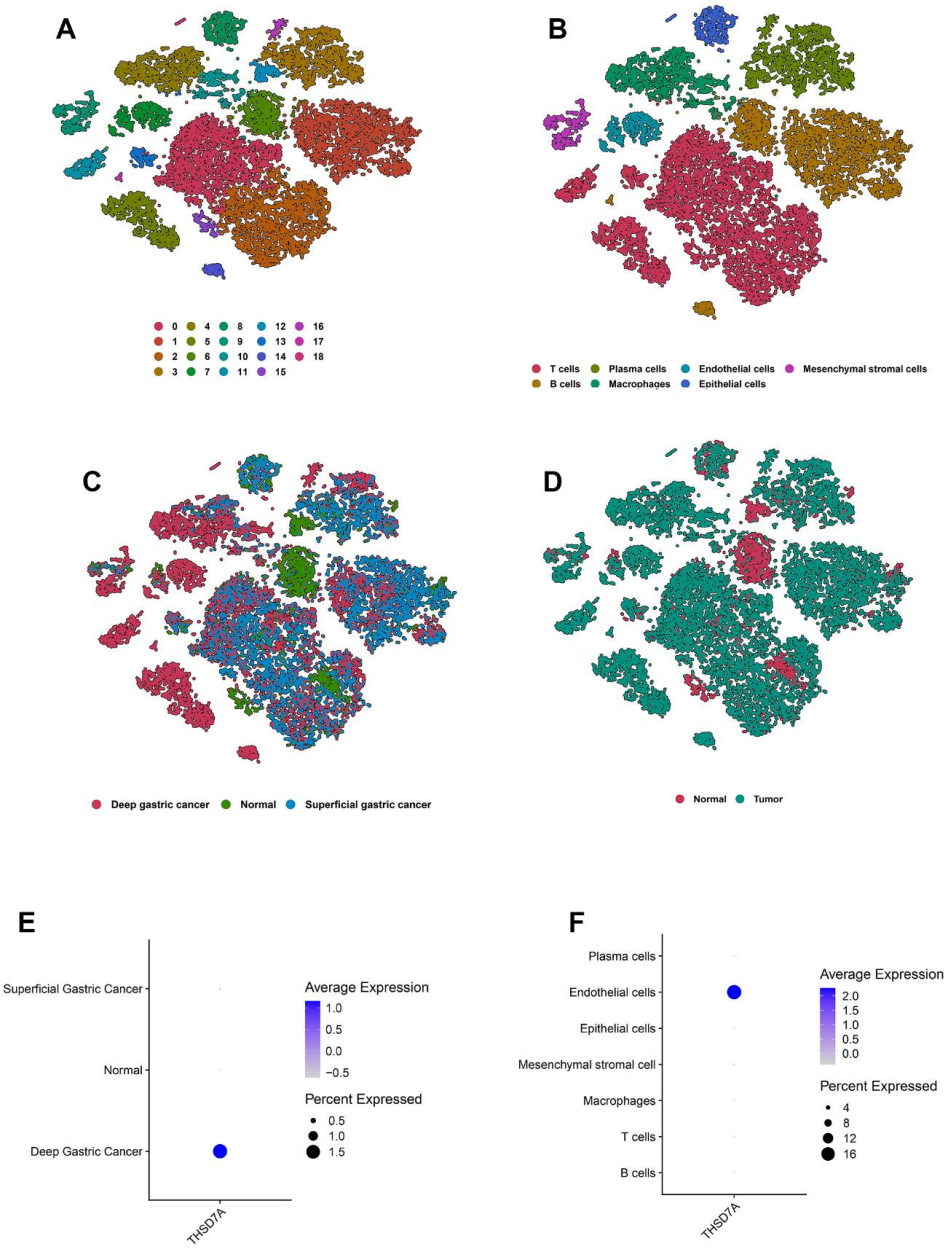
**Overview of the scRNA-seq data from 10 gastric cancer tissue samples and 4 normal gastric tissue samples.** The *t*-SNE plots classified by cluster (**A**), cell type (**B**), and tissue type (**C**, **D**). The expression of THSD7A in different tissues (**E**) and cell types (**F**).

After performing cell quality control (Supplementary Figure 2), we obtained a total of 19766 cells from 10 gastric cancer samples and 4 normal gastric tissue samples. We performed dimensionality reduction via principal component analysis and imported the first 9 principal components (Supplementary Figure 3) into the *t*-distributed Stochastic neighbor Embedding (*t*-SNE) algorithm; 19 cell subgroups were obtained via clustering (Figure 8A). T cells, plasma cells, endothelial cells, mesenchymal stromal cells, B cells, macrophages, and epithelial cells were characterized via annotation of the “CellMarker” database (Figure 8B). Marker genes of the cell subpopulations are shown (Supplementary Figure 4). Meanwhile, we visualized the cell subpopulations in different samples (Supplementary Figure 5) and different tissue types (Figure 8C, 8D). THSD7A expressed in deep gastric cancer tissues (Figure 8E) was mainly distributed in the endothelial cell subpopulation (Figure 8F). These findings suggested that THSD7A may be an endothelial cell-related gene.

### Trajectory analysis

**Figure 9:**
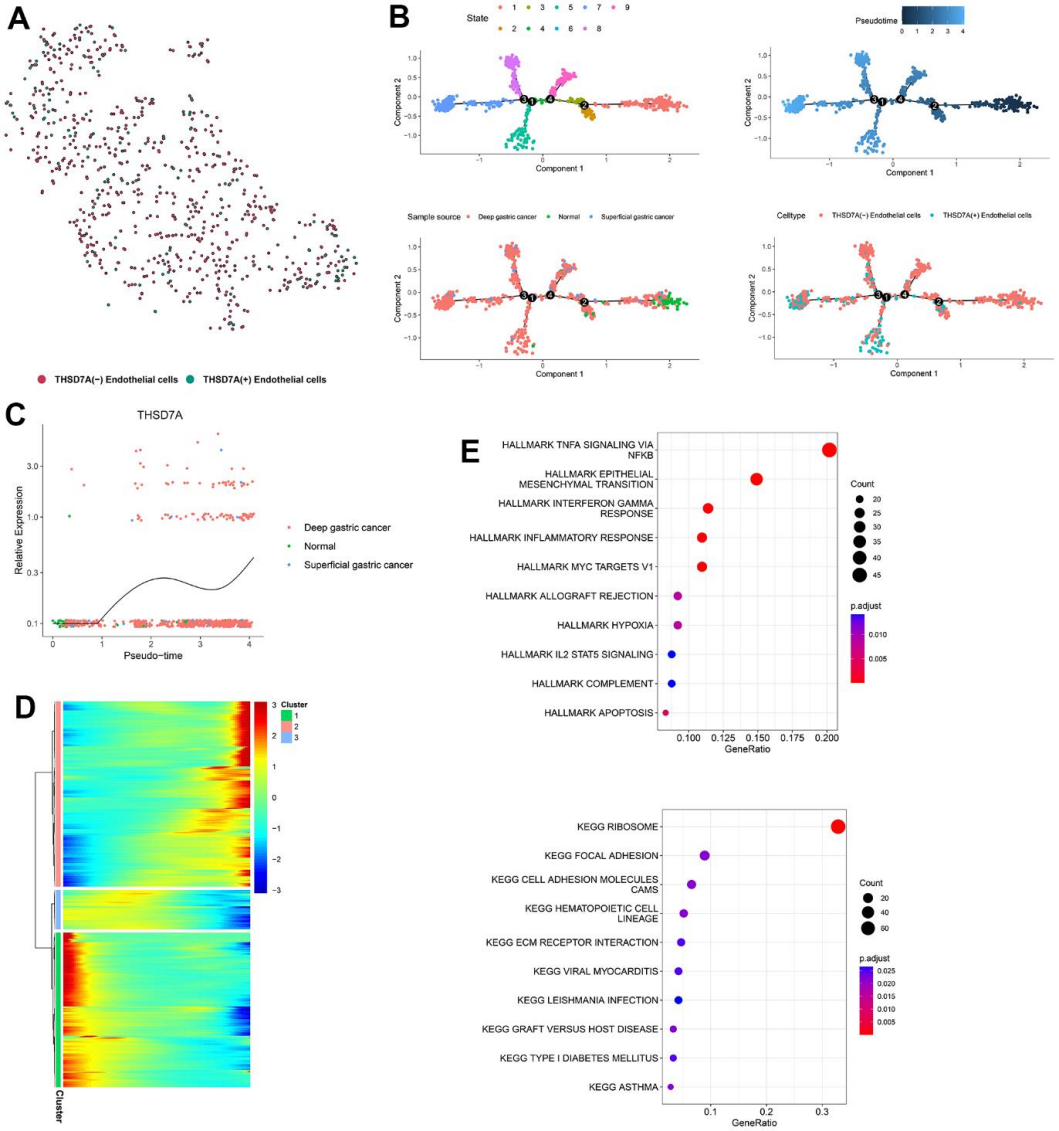
**Pseudotime analysis of endothelial cell subpopulations.** (**A**) Endothelial cells are classified into two subpopulations, namely THSD7A (+) and THSD7A (-). (**B**) Pseudotime trajectory of endothelial cells. (**C**) Change in THSD7A expression with pseudotime trajectory in different types of tissues. (**D**) Dynamic expression of genes in the pseudotime trajectory; red represents upregulated gene expression; blue represents downregulated gene expression. (**E**) Enrichment analysis of gene cluster 2.

To explore the developmental trajectory of THSD7A-related endothelial cells, we first isolated the endothelial cell subpopulations in gastric cancer and normal gastric tissues, including 802 endothelial cells. The “PercentageFeatureSet” function identified 135 and 677 endothelial cells that expressed or did not express THS7DA, respectively (Figure 9A). We simulated the trajectory of endothelial cells using the Monocle2 algorithm, which divided endothelial cells into seven differentiation states (Figure 9B). From the perspective of pseudotime trajectory, the endothelial cells from normal gastric tissue were mainly distributed at the beginning of the branch, whereas endothelial cells from superficial and deep gastric cancer were distributed at the middle and end of the differentiation trajectory, respectively. THSD7A (+) endothelial cells were enriched at the end of the differentiation trajectory, compared with the THSD7A (-) endothelial cells. THSD7A expression gradually increased with pseudotime trajectory (Figure 9C). We further clustered the genes expressed along the differentiation trajectory of endothelial cells into three clusters and plotted a heat map (Figure 9D), where THSD7A was located in cluster 2, which contained 607 genes (Supplementary Table 1). We performed enrichment analysis for the genes in cluster 2, and the results for some pathways, such as EMT, inflammatory responses, IL2-STAT5 signaling pathway, cell adhesion molecules, focal adhesion, and ECM-receptor interaction were consistent with previous GSEA results (Figure 9E). Among the pathways mentioned above, the genes in cluster 2 were most enriched in the EMT pathway, suggesting that at the single-cell level, EMT may be a key pathway through which THSD7A and its co-expressed genes promote gastric cancer progression.

### Cell-cell communication analysis

**Figure 10:**
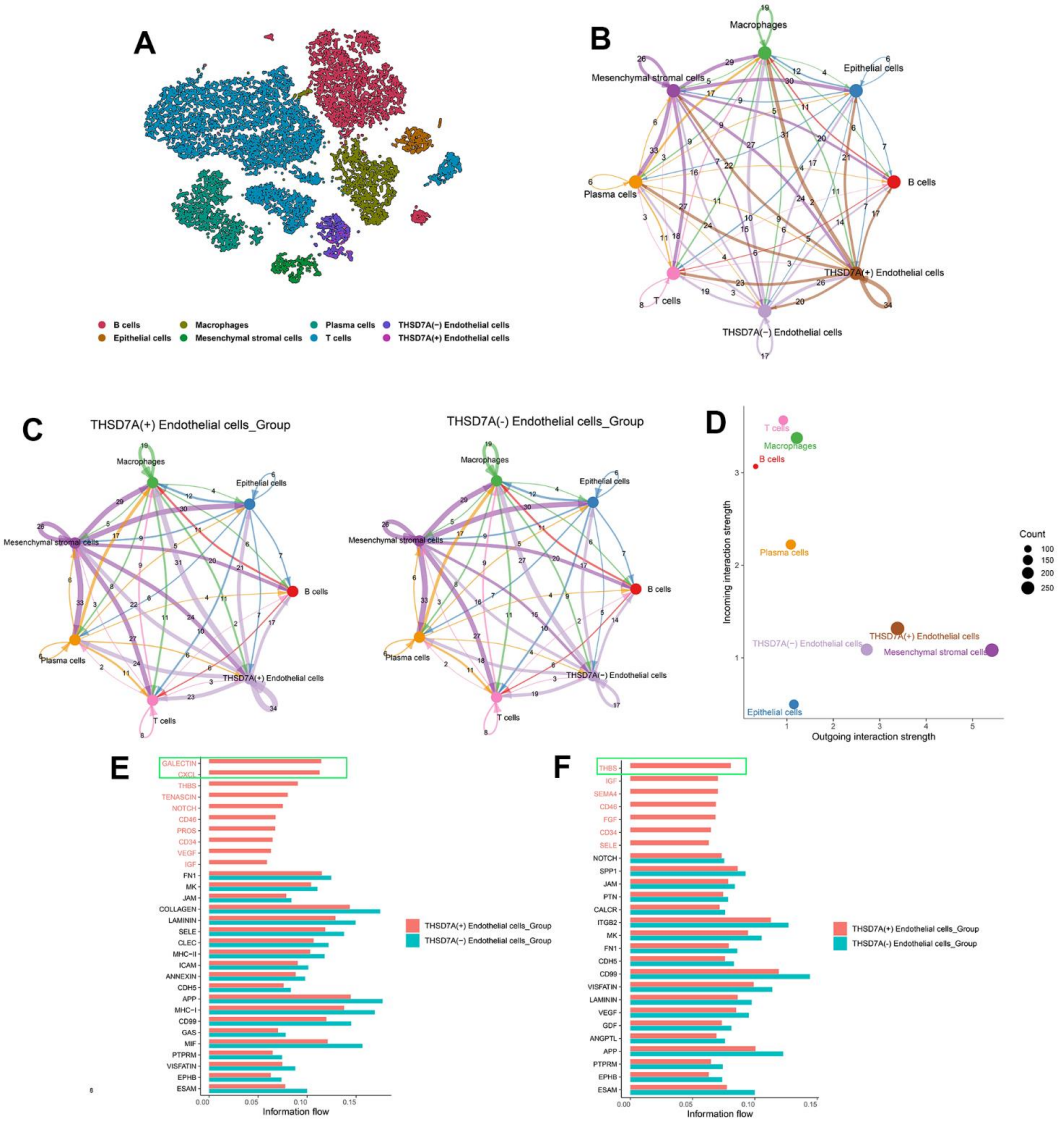
**The role of THSD7A-associated endothelial cells in intercellular communication.** (**A**) Isolation of gastric cancer-derived cell subpopulations and re-annotation of endothelial cell subpopulations. (**B**) Cellular communication of eight cell subpopulations, with the numbers representing the number of pathways. (**C**) Communication interactions between two THSD7A (+) endothelial cells and THSD7A (-) endothelial cells and other cell subpopulations, with the number representing the number of pathways. (**D**) Intensity and number of signals in each cell subpopulation. The values of the coordinate axes and the sizes of the circles represent the intensity and number of output and input signals, respectively. Differences in cellular communication between two groups when endothelial cells act as signal receivers (**E**) and senders (**F**).

In order to determine the interactions between THSD7A (+) endothelial cells and other cell subpopulations in the gastric cancer microenvironment, the cell subpopulations from gastric cancer tissue were isolated and re-annotated (Figure 10A). Subsequently, we conducted a cellular interaction analysis of the eight cell subpopulations that were obtained (Figure 10B) and compared the differences in communication interactions between both THSD7A (+) and THSD7A (-) endothelial cells and the other cells (Figure 10C). Our results indicated that as both signal senders and receivers, the THSD7A (+) endothelial cell subpopulation enriched a stronger signaling pathway network than the THSD7A (-) endothelial cell subpopulation (Figure 10D). We visualized the relationships among the ligand-receptor pairs in the relevant signaling pathways when THSD7A (+) and THSD7A (-) endothelial cells act as signal senders and receivers, respectively (Supplementary Figure 6). When endothelial cells were analyzed as signal senders, the signaling pathways that were only enriched in THSD7A (+) endothelial cells were those such as galectin, C-X-C motif chemokine ligand (CXCL), thrombospondin (THBS), and tenascin (Figure 10E); when endothelial cells were analyzed as signal receivers, the signaling pathways that were only enriched in THSD7A (+) endothelial cells were those such as THBS, insulin-like growth factor, and semaphorin-4A, with CD46 only being present in the THSD7A (+) endothelial cells (Figure 10F). Among them, the galectin (LGALS9 - CD44 and LGALS9 - CD45), CXCL (CXCL12 – [C-X-C chemokine receptor type 4] CXCR4) and THBS (THBS1 - CD47, THBS1 - SDC1, and THBS1 - SDC4) pathways showed the most significant communication differences in signal sending and receiving, respectively. Therefore, we visualized the intercellular communication relationships between these three pathways (Supplementary Figure 7). We also analyzed interactions within the endothelial cell subpopulation and found that THSD7A (+) and THSD7A (-) endothelial cells showed strong levels of interactions in the amyloid-beta precursor protein (APP)-CD74 signaling pathway (Supplementary Figure 8).

### Knocking down THSD7A inhibits the proliferation and migration of gastric cancer cells

**Figure 11:**
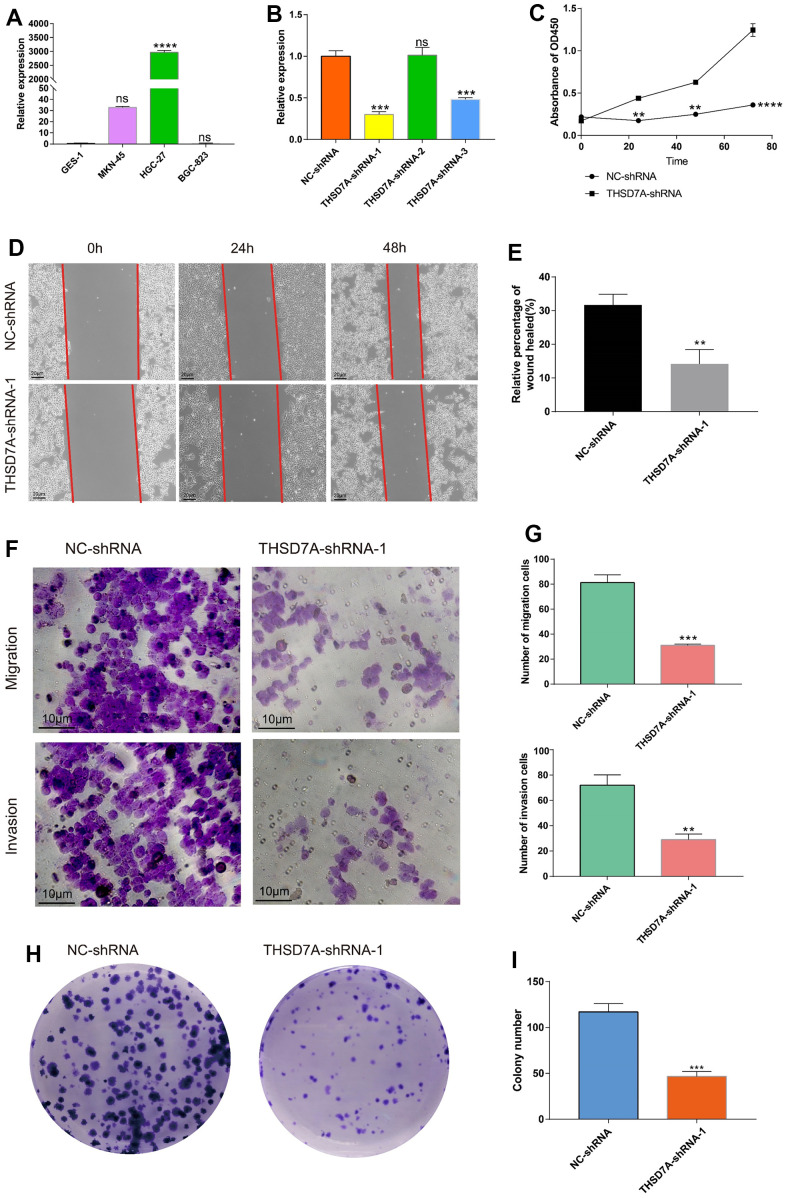
**Effects of THSD7A gene deletion on gastric cancer cell proliferation and invasion.** (**A**) The results of the qRT-PCR analysis of THSD7A expression in normal gastric cells and gastric cancer cells. (**B**) In HGC-27 cells, THSD7A expression is downregulated in the shRNA group, with the THSD7A-shRNA-1 group being downregulated most significantly. (**C**) CCK-8 assay results showing the proliferation and viability of HGC-27 cells following THSD7A knockout. (**D**, **E**) Wound-healing assay results showing the migration ability of HGC-27 cells following THSD7A knockout (scale bar = 20 μm). (**F**, **G**) Transwell assay results showing the invasion and migration abilities of HGC-27 cells in the NC-shRNA and THSD7A-shRNA groups (scale bar = 10 μm). (**H**, **I**) Colony formation assay results comparing the number of clones of HGC-27 cells between the NC-shRNA and THSD7A-shRNA groups (*: P<0.05, **: P<0.01, ***: P<0.001, ****:P<0.0001, ns: P>0.05. Error bars represent standard deviation). (GES-1 and NS-shRNA are the control group).

The effects exerted by THSD7A on the proliferation and migration of gastric cancer cells were investigated. qRT-PCR analysis showed that THSD7A was significantly upregulated in gastric cancer cells (MKN-45 and HGC-27) compared with normal gastric cells, with THSD7A being most significantly expressed in the HGC-27 cells (Figure 11A). We selected HGC-27 cells for subsequent functional experimental analysis and used various types of short hairpin RNAs (shRNAs) to suppress THSD7A expression in the HGC-27 cells. The qRT-PCR test revealed that THSD7A-shRNA-1 had the best silencing efficiency (Figure 11B). Cell counting kit-8 (CCK-8) assay results showed that, following THSD7A gene knockout, the number of HGC-27 cells were significantly reduced compared to that in the normal control (NC)-shRNA group (Figure 11C). The results of the wound scratch assay indicated that the healing ability of gastric cancer cells was reduced at different periods following THSD7A gene knockout (Figure 11D, 11E). The results of the transwell assay showed that the migration and invasion abilities of HGC-27 cells were significantly reduced following THSD7A gene knockout (Figure 11F, 11G). The result of the colony formation assay showed that THSD7A deletion significantly reduced the number of new HGC-27 cell clones (Figure 11H, 11I).

## DISCUSSION

With due consideration given to the low survival rate of those afflicted with gastric cancer, it is necessary to explore effective gastric cancer-related biomarkers for prognostic value and usefulness as new therapeutic targets. The mechanism of action and the diagnostic value of THSD7A in gastric cancer have not been fully investigated. Therefore, we performed a comprehensive analysis of the bulk and single cell RNA sequencing (scRNA-seq) data pertaining to gastric cancer in public databases. An examination of bulk sequencing data from the TCGA-STAD and GEO databases indicated that THSD7A was highly expressed in gastric cancer tissues compared to non-cancerous tissue samples. Furthermore, THSD7A expression was correlated with TNM stage, grade, race, and histological subtypes. Survival analysis, conducted using the GEPIA database and the Kaplan–Meier Plotter tool, showed that high THSD7A expression predicted shorter OS, DFS, FPS, and PPS in patients with gastric cancer, which further supported the finding that the upregulation of THSD7A was correlated with the degree of gastric cancer malignancy. Univariate and multivariate Cox regression analyses indicated that THSD7A was an independent risk factor for gastric cancer. To determine the efficacy of THSD7A as a clinical application, a THSD7A-nomogram was drawn in combination with clinical features, and its calibration curves reflected good predictive performance.

The functional enrichment analysis revealed that THSD7A expression in gastric cancer may be associated with pathways such as inflammatory responses, angiogenesis, EMT, focal adhesion, and cell adhesion molecules. Ma et al. reported that while inflammatory mediators secreted by immune cells promoted EMT on the one hand, they transformed normal gastric epithelial cells to cancer cells on the other hand, by increasing cellular DNA damage and mutations [25]. Uncontrolled angiogenesis is considered a hallmark of cancer [26]. Previous studies have shown that EMT not only regulates the invasion and metastasis of tumor cells [27], but also facilitates the development of multidrug resistance [28]. Furthermore, the dysregulation of ECM homeostasis and cell-cell adhesion in the TME is a key driver of cancer development [29–31]. Increased activity of the IL2-STAT5 signaling pathway was seen in gastric cancer patients with elevated levels of THSD7A expression. The research done by Lutz et al. unveiled that the occurrence of CD8+ T-cell exhaustion in pancreatic cancer is regulated by the IL2-STAT5 signaling pathway [32]. Chronic inflammation is a significant factor in the pathogenesis of several malignancies, as the inflammatory elements inside the TME promote interaction between immune and non-immune cells, so creating a conducive environment for the advancement of cancer [33]. Eke et al. have previously reported that the focal adhesion signalling pathway functions as an intermediary mechanism for drug resistance in tumor cells [34]. Considered together, these findings suggest that the contribution of THSD7A to the development of gastric cancer cell invasion and drug resistance is multi-dimensional.

The presence of TICs in the TME has been clearly identified as an important prognostic indicator of patient survival as well as a potential target in the treatment of tumors [35]. To the best of our knowledge, our current study is the first to report the relationship between THSD7A and TICs. Upregulation of THSD7A showed a significantly positive correlation with resting mast cells, M2 macrophages, and Tregs, but showed a negative correlation with follicular helper T cells, activated CD4^+^ memory T cells, and M1 macrophages, suggesting that the upregulation of THSD7A promotes the recruitment of resting mast cells, M2 macrophages, and Tregs in the microenvironment of gastric cancer. The TME is enriched in mast cells due to recruitment by chemokines, which promote the EMT of tumor cells via the secretion of CXCL8 [36]. Angiogenic factors, which promote tumor angiogenesis, are produced under cytokine stimulation and hypoxic environments [37, 38]. Ribatti et al. reported that mast cells were highly correlated with gastric cancer angiogenesis [39]. Evidently, the massive infiltration of M2 macrophages that act as immunosuppressive cells in the TME, predicts a shorter survival time [40]. According to Tanaka et al., Tregs heavily infiltrate the TME of a variety of human tumors, such as gastrointestinal tumors, lung cancer, and breast cancer, where they mediate immunosuppression via CTLA4. Tregs are key cells that trigger tumor immune escape [41]. In their study, Cui et al. discovered that T follicular helper cells possess the ability to engage in interactions with tumor-specific B cells, hence facilitating the manifestation of anti-tumor immune responses [42]. Prior research has indicated that individuals afflicted with tumors that have a high degree of infiltration by activated CD4+ memory T cells and plasma cells tend to experience a favorable prognosis [43, 44]. Exosomes generated from M1 macrophages exhibit a specific affinity for the interleukin-4 receptor found on M2-polarized macrophages. Consequently, this interaction leads to the suppression of tumor development [45]. The M1 macrophage exhibits promising potential for the therapeutic intervention of several types of malignancies [46, 47]. Furthermore, NK cells are acknowledged as immune cells that have anti-tumor properties [48]. This study found that the expression of THSD7A may be associated with the suppression of the anti-tumor immune response.

Compared with surgery alone, adjuvant chemotherapy has improved the survival rate of patients with gastric cancer. However, many patients with gastric cancer do not benefit from adjuvant chemotherapy [49], and the therapeutic response to chemotherapeutic agents is affected by the interaction between cells in the TME and the chemokine network produced by them [50]. The currently used conventional tumor staging systems are not ideal for predicting the response to chemotherapy [51], whereas biomarkers have shown the capacity to play a useful role in predicting the therapeutic response. Jiang et al. constructed a risk signal based on periostin, cyclooxygenase-2, and TME-associated cells that predicts the chemosensitivity of patients with gastric cancer [52]. The current study evaluated the IC_50_ values of selected chemotherapeutic agents in patients with gastric cancer in high- and low-THSD7A expression groups. Moreover, 5-fluorouracil and cisplatin are known important anti-cancer agents that are used against gastric cancer [53]. We found that upregulation of THS7DA may promote resistance to 5-fluorouracil and cisplatin in patients with gastric cancer. Further analysis indicated that sensitivity to bleomycin, doxorubicin, etoposide, and gemcitabine may be reduced in patients with high THSD7A expression. A previous enrichment analysis has shown that THSD7A is involved in resistance-related pathways, which may explain the decreased sensitivity shown by patients with high THSD7A expression to the above chemotherapeutic agents. Notably, upregulation of THSD7A increased sensitivity to lapatinib and pazopanib. A clinical trial targeting patients with human epidermal growth factor receptor 2 (HER2)-amplified gastroesophageal adenocarcinoma showed that the addition of lapatinib significantly prolonged patient survival compared with a combination chemotherapy consisting only of capecitabine and oxaliplatin [54]. In addition, lapatinib combined with paclitaxel resulted in significantly longer median OS in patients with HER2-amplified gastric cancer than paclitaxel alone [55]. Kim et al. reported that pazopanib inhibited the proliferation of fibroblast growth factor receptor 2-amplified gastric cancer cells [56]. Högner et al. found that pazopanib combined with 5-fluorouracil and oxaliplatin (FLO) was more effective than FLO alone in improving OS in patients with advanced gastric cancer [57]. However, the relationship between the above chemotherapeutic agents and THSD7A needs to be verified in future clinical trials. Immunotherapy has been widely used as an innovative approach in the treatment of solid tumors, and gastric cancer-related immune targets are being developed [58]. This study found that immune checkpoints, including HAVCR2, PDCD1LG2, TIGIT, and CTLA4, are highly expressed in patients with high expression of THSD7A. Current analyses combined with previous immune infiltration analyses, indicate that THSD7A not only induces a poor immune microenvironment, but also enables immune escape in gastric cancer. These results provide a clue for the development of immunotherapy targeting patients in the high THSD7A expression group.

The scRNA-seq technology compensates for traditional bulk sequencing defects, including the inability to reveal heterogeneity between cells, satisfies the requirements of various types of tumor research, and demonstrates the molecular characteristics of the TME in different pathological backgrounds by plotting maps for cancer cell subpopulations [59]. We analyzed the role played by THSD7A at the cell subpopulation level using scRNA-seq data. First, THSD7A was mainly expressed in endothelial cells with gastric cancer and minimally expressed in the endothelial cells of normal gastric tissues. Kuo et al. reported that soluble THSD7A is an N-glycoprotein with the ability to promote endothelial cell migration and accelerate angiogenesis via a mechanism that may be associated with the regulation of focal adhesions [60]. Our GSEA results showed that THSD7A participates in signaling pathways related to angiogenesis, focal adhesion, and cell adhesion molecules, which is consistent with the findings of previous studies. Subsequently, cell trajectory analysis revealed that THSD7A (+) endothelial cells are mainly located at the end of the differentiation trajectory, and that THSD7A expression increased with the progression of gastric cancer infiltration. These findings indicated that the upregulation of THSD7A may drive the process of gastric cancer cell invasion from a superficial level to a higher level. In addition, we analyzed the genes that changed with the differentiation trajectory and found that the expression pattern of genes in cluster 2 was consistent with changes in THSD7A. Enrichment analysis revealed that these genes mainly participated in pathways such as EMT, inflammatory responses, IL2-STAT5 signaling pathway, cell adhesion molecules, focal adhesion, and ECM-receptor interaction. Further analysis indicated that EMT mediated by THSD7A (+) endothelial cells may be an important pathway affecting gastric cancer cell proliferation and metastasis.

Intercellular communication analysis indicated that THSD7A (+) endothelial cells participated in a more unique signaling pathway network compared to THSD7A (-) endothelial cells. Among these pathways and ligand-receptor pairs, the galectin (LGALS9 - CD44 and LGALS9 - CD45), CXCL (CXCL12 – CXCR4), and THBS (THBS1 - CD47, THBS1 - SDC1 and THBS1 - SDC4) pathways differed significantly. Previous studies have reported that the differentiation and maintenance of Tregs are highly correlated with LGALS9-CD44 in the galectin signaling pathway [61]. Iqbal et al. found that endothelial cells recruit neutrophils by expressing LGALS9 ligands that bind to CD44 receptors, which in turn induce an inflammatory response [62]. LGALS9 may also inhibit the activation of B cells by binding to CD45 [63]. The CXCL signaling pathway not only plays a key role in gastric cancer immune resistance [64], but also induces EMT progression vis the CXCL12-CXCR4 axis [65], the role of which has also been confirmed in breast cancer metastasis [66]. THBS1 and CD47 also jointly participate in anti-tumor immunosuppressive effects [67], where upregulated expression of THBS1-CD47 not only accelerates the progression of EMT, but also induces fibrosis and inflammation [68]. The interaction between THBS1 and SDC1 expressed in malignant gliomas promotes tumor cell invasion [69]. Ferrari do Outeiro-Bernstein et al. discovered that the binding of THBS1 to SDC4 inhibited apoptosis of vascular endothelial cells and maintained their adhesion function [70]. Yao et al. reported that SDC1 and SDC4 were upregulated in lung adenocarcinoma cells with high EMT scores [71]. Moreover, THSD7A (+) and THSD7A (-) endothelial cells may interact within endothelial cell subpopulations in an autocrine manner, with APP-CD74 playing a key role. Although APP is an Alzheimer’s disease-associated protein, it is also reportedly overexpressed in nasopharyngeal, colon, and pancreatic cancers [72–74]. The upregulation of CD74, which acts as a key signal in the immune process, is associated with poor prognoses, as has been demonstrated in gastric cancer, pancreatic cancer, and glioma [75–77]. Borghese et al. reported that CD74 may be a potential target in the treatment of tumors [78]. The role of APP-CD74 in cancer has rarely been reported and requires further investigation. This study demonstrated that the upregulation of THSD7A may drive immunosuppression, inflammatory responses, and activation of EMT signaling pathways via the aforementioned ligand-receptor pairs, resulting in gastric cancer progression.

Previous studies have confirmed that inhibition of THS7DA may significantly inhibit the proliferative and invasive capacities of esophageal cancer cells [79]. Our cellular experiments demonstrated that THSD7A expression was significantly higher in HGC-27 cells than in GES-1 cells, and that silencing it significantly inhibited the proliferation, invasion, and migration abilities of HGC-27 cells.

The present study observed that the expression of THSD7A was notably higher in HGC-27 cells. Furthermore, we conducted a preliminary investigation to examine the impact of inhibiting THSD7A on the activity of gastric cancer cells. Nevertheless, it is important to acknowledge certain limitations in our study. Firstly, due to financial constraints, we were only able to conduct functional experiments on HGC-27 cells. Consequently, the findings may not comprehensively represent the impact of inhibiting THSD7A on gastric cancer. Furthermore, we anticipate investigating the protein-level expression levels of THSD7A in gastric cancer cells, as well as its interference in mice tumor models. These future experiments should employ more advanced and established experimental equipment, as well as more refined experimental techniques. In addition, we intend to carry out clinical prospective research including individuals diagnosed with stomach cancer.

In conclusion, the findings of this study revealed that upregulation of THSD7A expression in endothelial cells is associated with the recruitment of tumor-associated immunosuppressive cell infiltration as well as poor prognoses in patients with gastric cancer. Furthermore, our findings indicate that THSD7A shows potential for use as an effective prognostic biomarker for gastric cancer and a new target in the treatment of gastric cancer.

## Supplementary Material

Supplementary Figures

Supplementary Table 1
