# Supplementary material for: Integrated analysis of single-cell and bulk RNA-sequencing data reveals the prognostic value and molecular function of THSD7A in gastric cancer
Source: Aging (Albany NY). 2023 Oct 30;15(21):11940–69. doi: 10.18632/aging.205158 (PMC10683630; doi:10.18632/aging.205158)
Supplement: Supplementary Figures [file aging-15-205158-s001.pdf]

SUPPLEMENTARY FIGURES

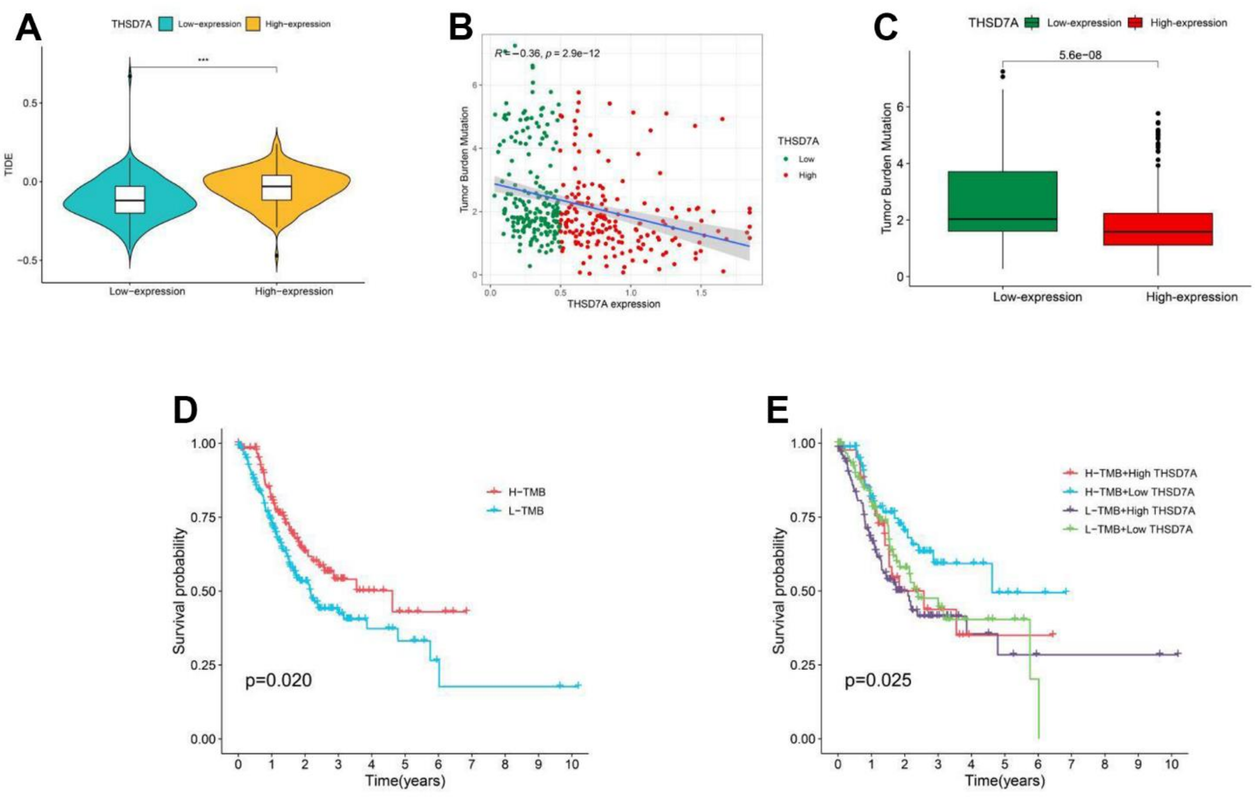

Supplementary Figure 1. Evaluation of immunotherapy efficacy and tumor mutation burden. (**A**) Evaluation of immunotherapy effectiveness in individuals with high- and low-THSD7A expression. (**B**) Correlation between THSD7A expression and TMB. (**C**) Distribution of TMB scores in high and low THSD7A groups (H: High; L: Low). (**D**) Gastric cancer patients in high- and low-TMB groups have different survival rates. (**E**) Patients with various levels of THSD7A expression and TMB scores had variable survival rates for gastric cancer (*: P<0.05, **: P<0.01, ***: P<0.001, ns: P>0.05.).

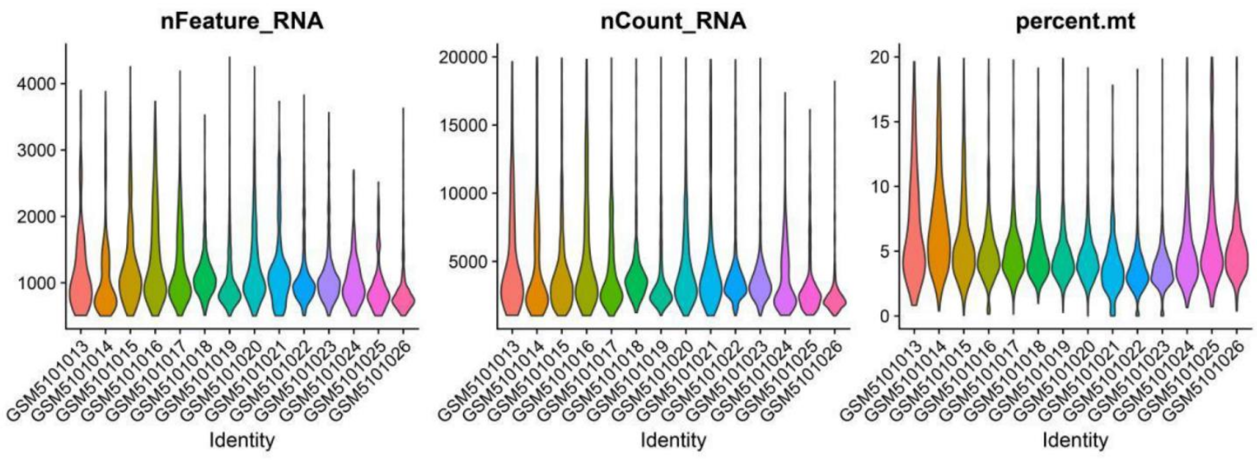

Supplementary Figure 2. Quality control of scRNA-seq data.

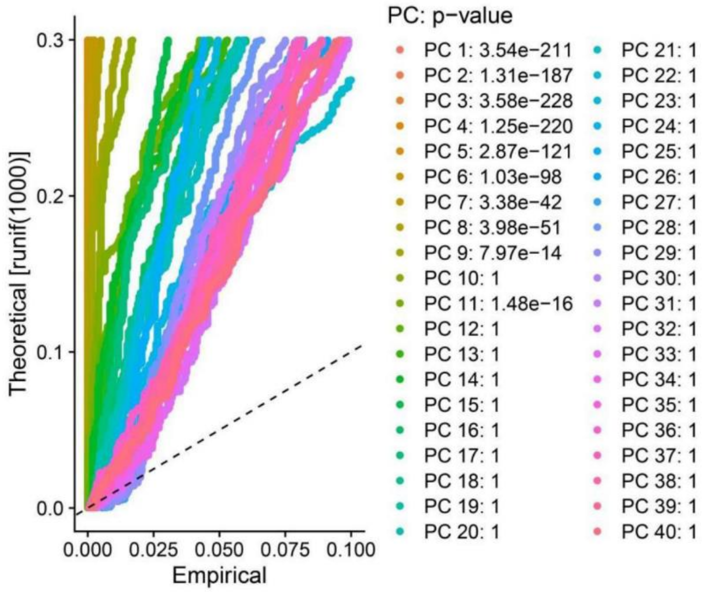

Supplementary Figure 3. First nine principal components (PCs) obtained from principal component analysis (PCA) dimensionality reduction analysis.

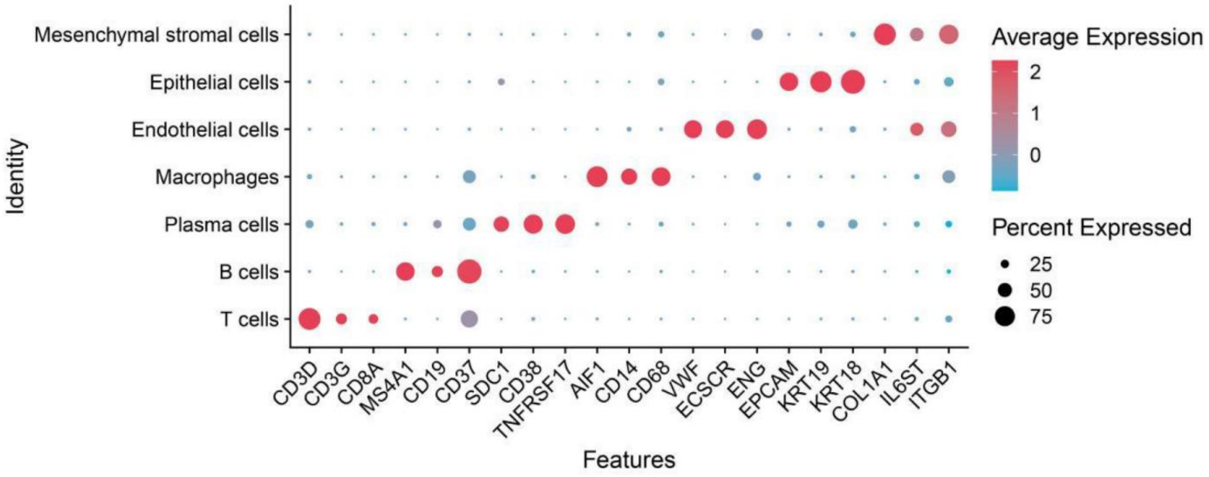

Supplementary Figure 4. Marker genes corresponding to seven cell subpopulations.

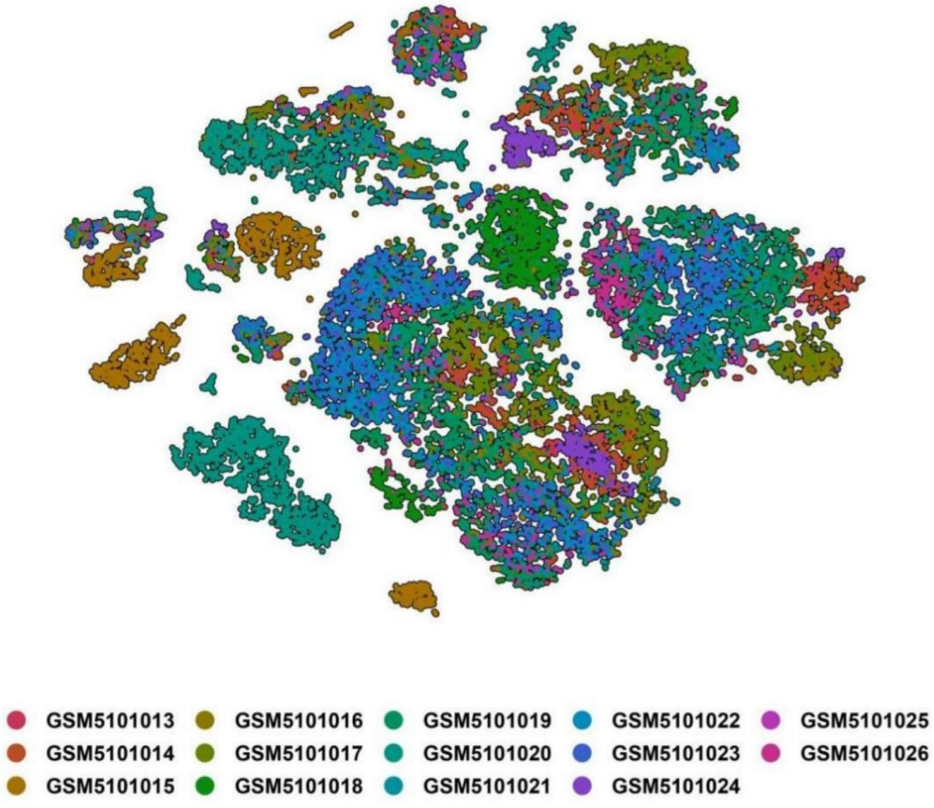

Supplementary Figure 5. The t-SNE plots classified by the source of samples.

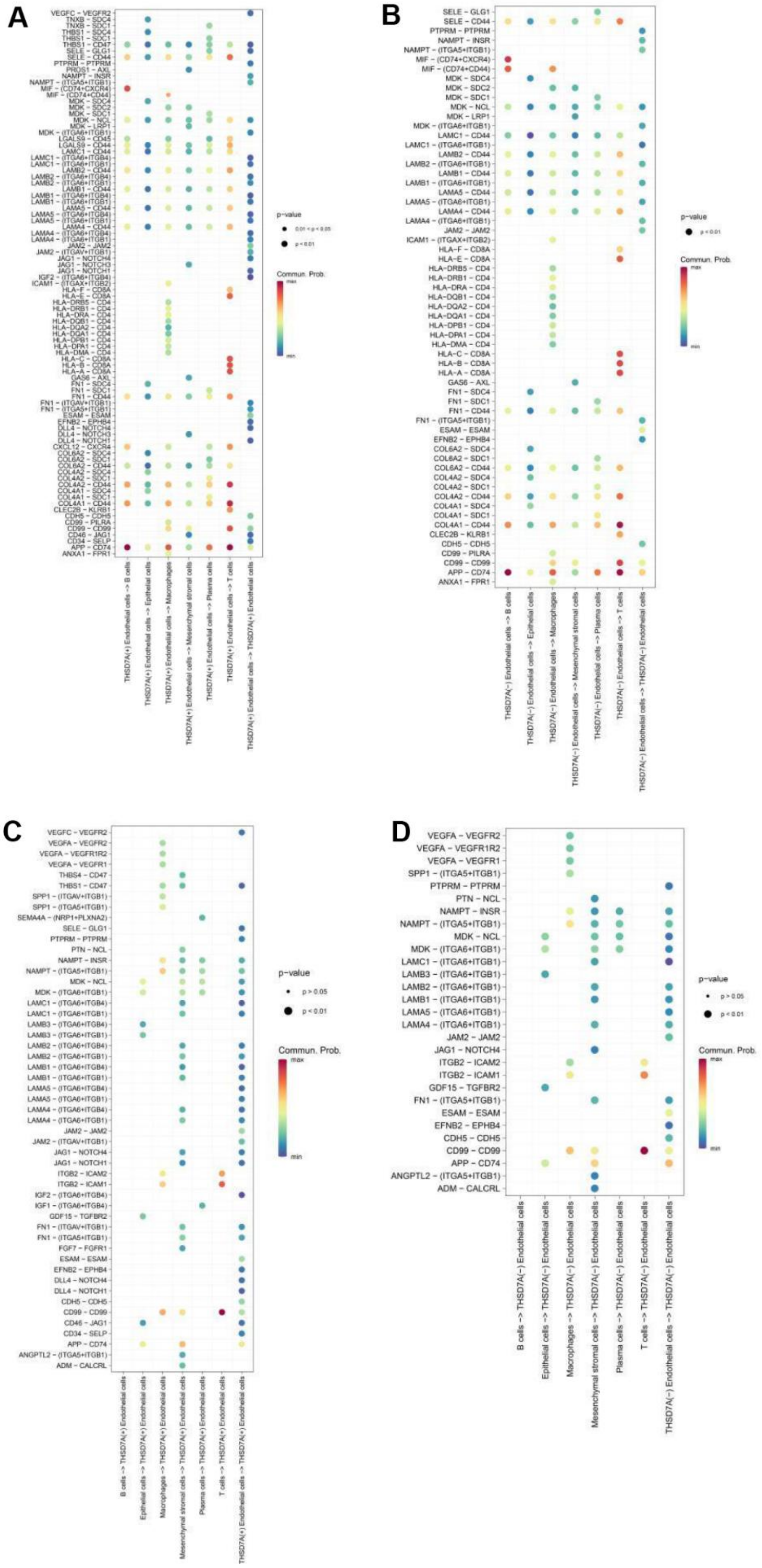

Supplementary Figure 6. Communication interactions between endothelial cells and other cell subpopulations when the endothelial cells act as signal senders (**A**, **B**) and receivers (**C**, **D**).

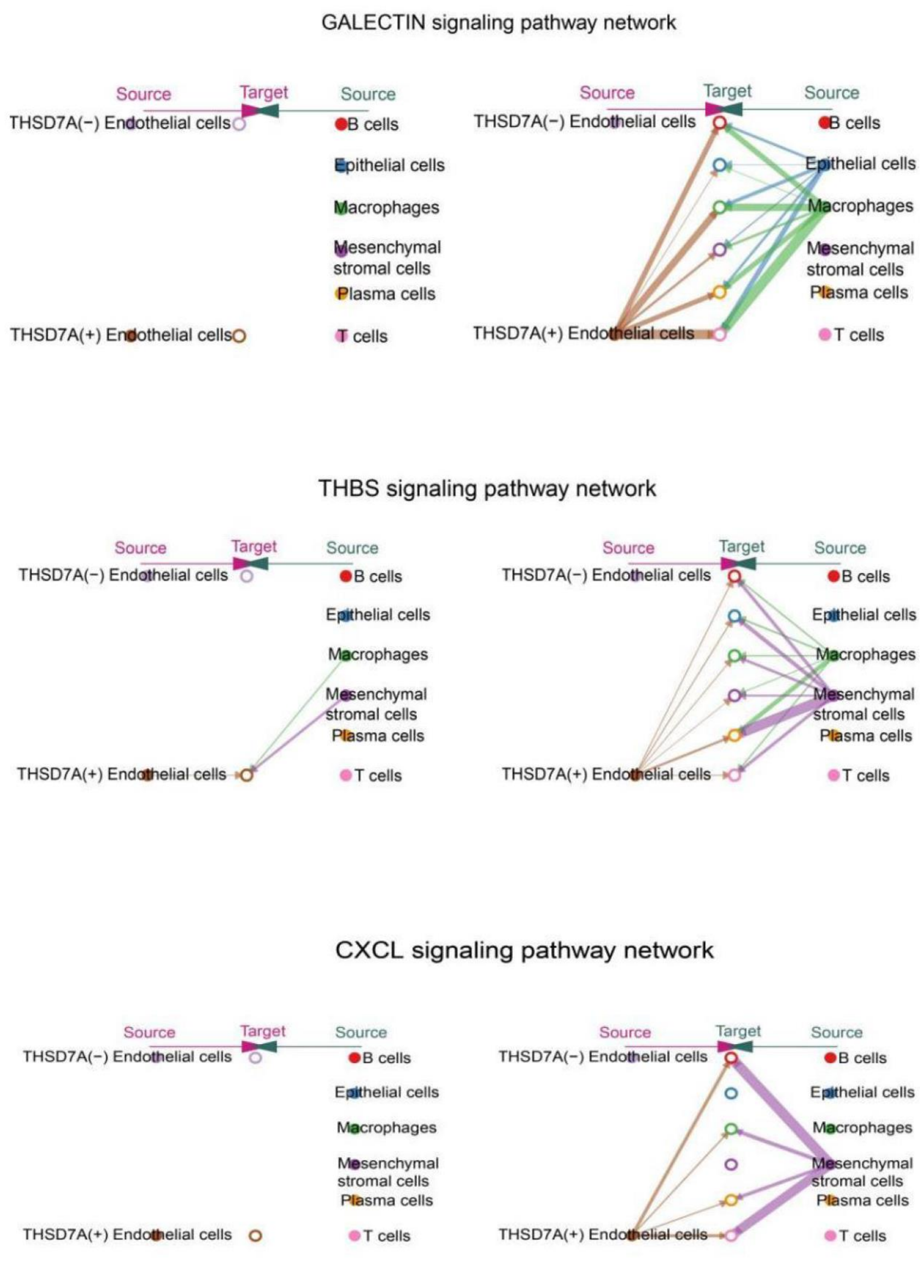

Supplementary Figure 7. Intercellular communication among galectin, CXCL and THBS signalling pathways.

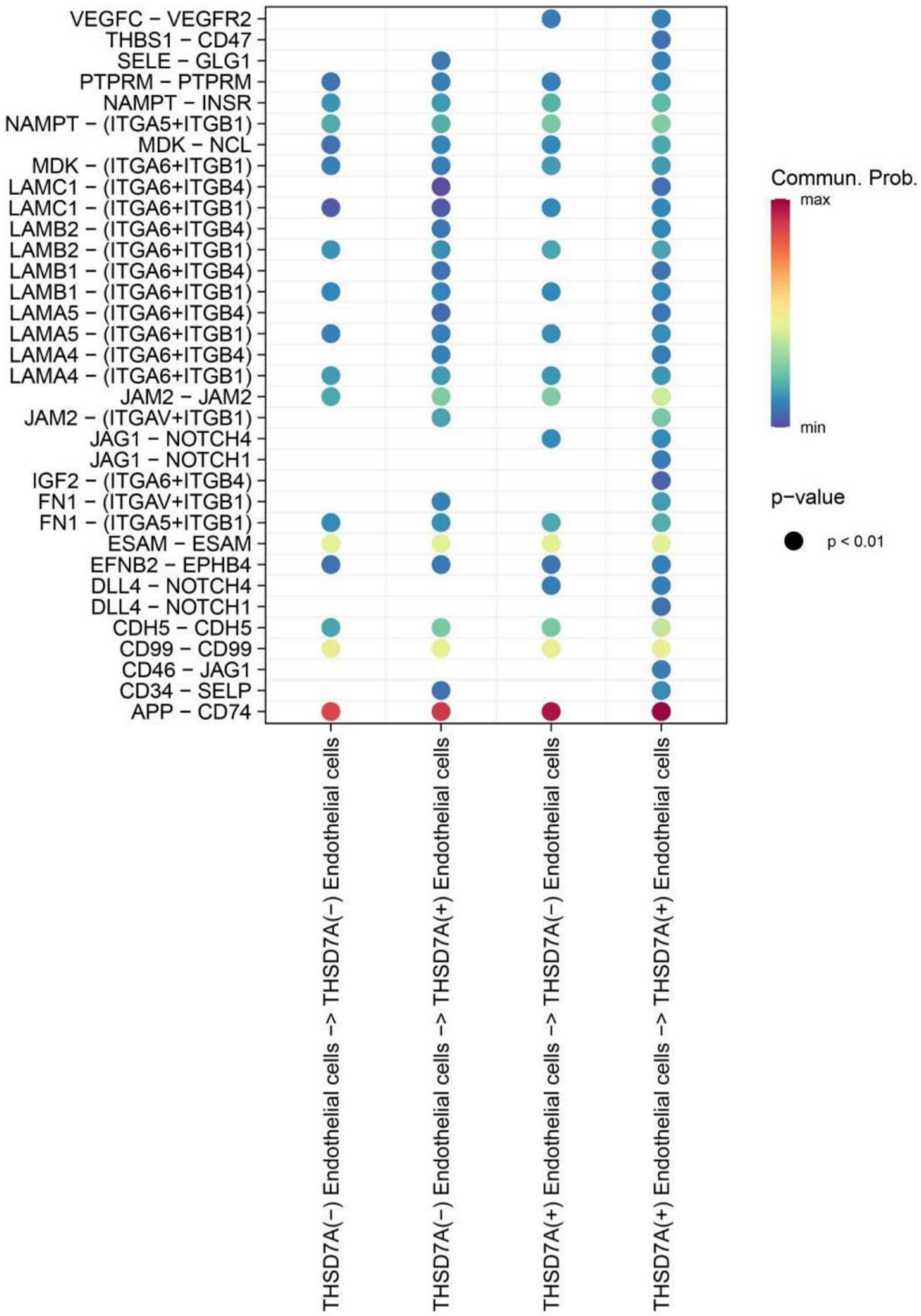

Supplementary Figure 8. Cellular interactions within endothelial cell subpopulations.
